# Hypertension-gut microbiota research trends: a bibliometric and visualization analysis (2000–2025)

**DOI:** 10.3389/fmicb.2025.1543258

**Published:** 2025-08-12

**Authors:** Qilin Chen, Chunzhen Ren, Chang Shu, Xue Yang, Hugang Jiang, Xiaodong Zhi, Chunling Wang, Kai Liu, Xinke Zhao, Yingdong Li

**Affiliations:** ^1^School of Traditional Chinese and Western Medicine, Gansu University of Chinese Medicine, Lanzhou, China; ^2^Gansu Province Key Laboratory of Chinese Medicine for the Prevention and Treatment of Chronic Diseases, Lanzhou, China; ^3^Department of Traditional Chinese Medicine, KweiChow Moutai Hospital, Renhuai, China; ^4^Key Clinical Specialty of the National Health Commission of the People’s Republic of China, Key Specialized Cardiovascular Laboratory National Administration of Traditional Chinese Medicine, Lanzhou, China; ^5^School of Clinical Medicine, Gansu Medical College, Pingliang, China

**Keywords:** hypertension, gut microbiota, bibliometric study, CiteSpace, VOSviewer, research trends, cutting-edge trends Workflow

## Abstract

**Background:**

Hypertension is a major global public health challenge affecting over 1.3 billion people. Emerging evidence indicates that gut microbiota regulates blood pressure through metabolic and immune-inflammatory pathways. This provides novel insights into hypertension mechanisms and facilitates targeted interventions. However, research in this field faces three major challenges: (1) fragmented knowledge, (2) limited clinical translation, and (3) unclear developmental trajectories. Consequently, conventional reviews cannot adequately capture its dynamic evolution.

**Objective:**

Using publications from the Web of Science Core Collection (2000–2025), we conducted a bibliometric analysis with CiteSpace and VOSviewer to map collaborative networks, analyze research hotspot evolution, identify emerging frontiers, and provide quantitative insights for field advancement.

**Methods:**

We retrieved 2,827 qualified publications through Boolean logic search, then performed analyses including: annual publication trends, national/institutional/author collaboration networks, keyword co-occurrence and clustering, burst detection, and timeline/mountain range visualizations using CiteSpace and VOSviewer.

**Results:**

Publication trends evolved through three phases: initial accumulation (annual output <50), accelerated growth, and stable maturation (250–450 annually). Driven by technology and clinical needs, China (918) and the US (676) led research, with networks involving Italy, Spain, etc. Academic institutions like the Univ. of Florida and Zhejiang Univ. were pivotal, and key teams (e.g., Yang Tao, Raizada Mohan K.) focused on mechanisms and translation. Research hotspots centered on “gut microbiota” and “blood pressure,” forming three modules: metabolic regulation, complication associations, and intervention strategies. Cluster analysis identified 10 groups—including short-chain fatty acids and TMAO—spanning basic to clinical research. Post-2017 foci like *Akkermansia muciniphila*, Mendelian randomization, and pulmonary hypertension signal a shift to precision mechanisms and personalized interventions.

**Conclusion:**

This study establishes a quantitative analytical framework for hypertension-gut microbiota research, revealing a collaborative landscape led by China and the United States with multidisciplinary integration. We identify metabolic reprogramming and microbiota-targeted interventions as core research priorities, providing theoretical foundations to address clinical translation barriers and advance precision medicine. Future research should strengthen cross-disciplinary collaboration, prioritize investigation of ethnicity-specific microbial signatures and microbiota-drug interactions, and accelerate clinical translation of targeted therapies.

## Graphical abstract

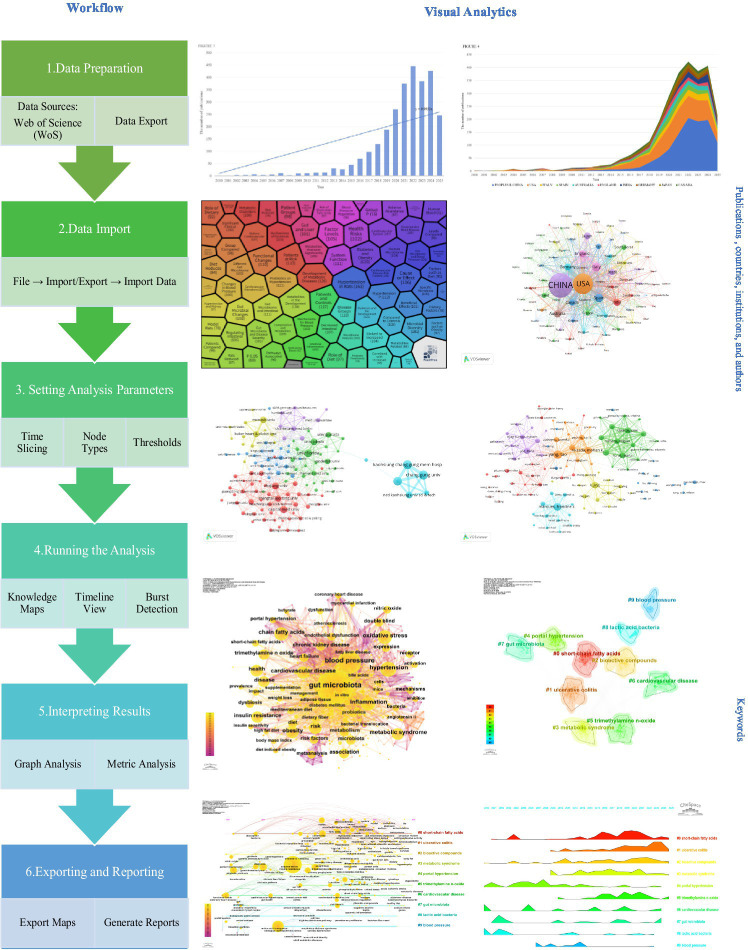

## 1 Introduction

Hypertension represents the leading global public health threat, affecting over 1.3 billion people worldwide and serving as a primary driver of cardiovascular disease and renal failure. Despite significant advances in conventional therapeutic strategies, including renin-angiotensin-aldosterone system inhibitors and calcium channel blockers, approximately 30% of patients present with treatment-resistant hypertension, indicating fundamental gaps in our understanding of disease etiology (Carey et al., 2018; NCD Risk Factor Collaboration, 2021).

Recent breakthroughs in gut microbiota research have revealed profound connections with hypertension. The microbiota influences blood pressure regulation through multiple pathways, including short-chain fatty acid metabolism, bile acid metabolism, and immune-inflammatory modulation, offering novel perspectives for understanding hypertensive mechanisms and implementing precision interventions. Since Pluznick and colleagues first reported in Nature (2013) the mechanisms by which gut microbial metabolites regulate host blood pressure, interdisciplinary research on hypertension and microbiota has experienced rapid expansion (Pluznick et al., 2013). By 2024, the field has generated over 5,000 publications across three major research directions: mechanistic studies investigating how microbial compositional differences influence metabolite production and subsequently alter vascular endothelial function; clinical translational research including randomized controlled trials of probiotic or prebiotic interventions; and technology-driven studies integrating multi-omics data with artificial intelligence predictive models. However, three core challenges persist: knowledge fragmentation, with research themes scattered across microbiology, cardiovascular medicine, and nutrition without systematic integration; prominent translational bottlenecks, as current research relies predominantly on animal studies (>70%), resulting in relatively weak clinical evidence chains; and unclear evolutionary pathways, lacking systematic evaluation of emerging technologies such as phage therapy and fecal microbiota transplantation.

**Figure 1:**
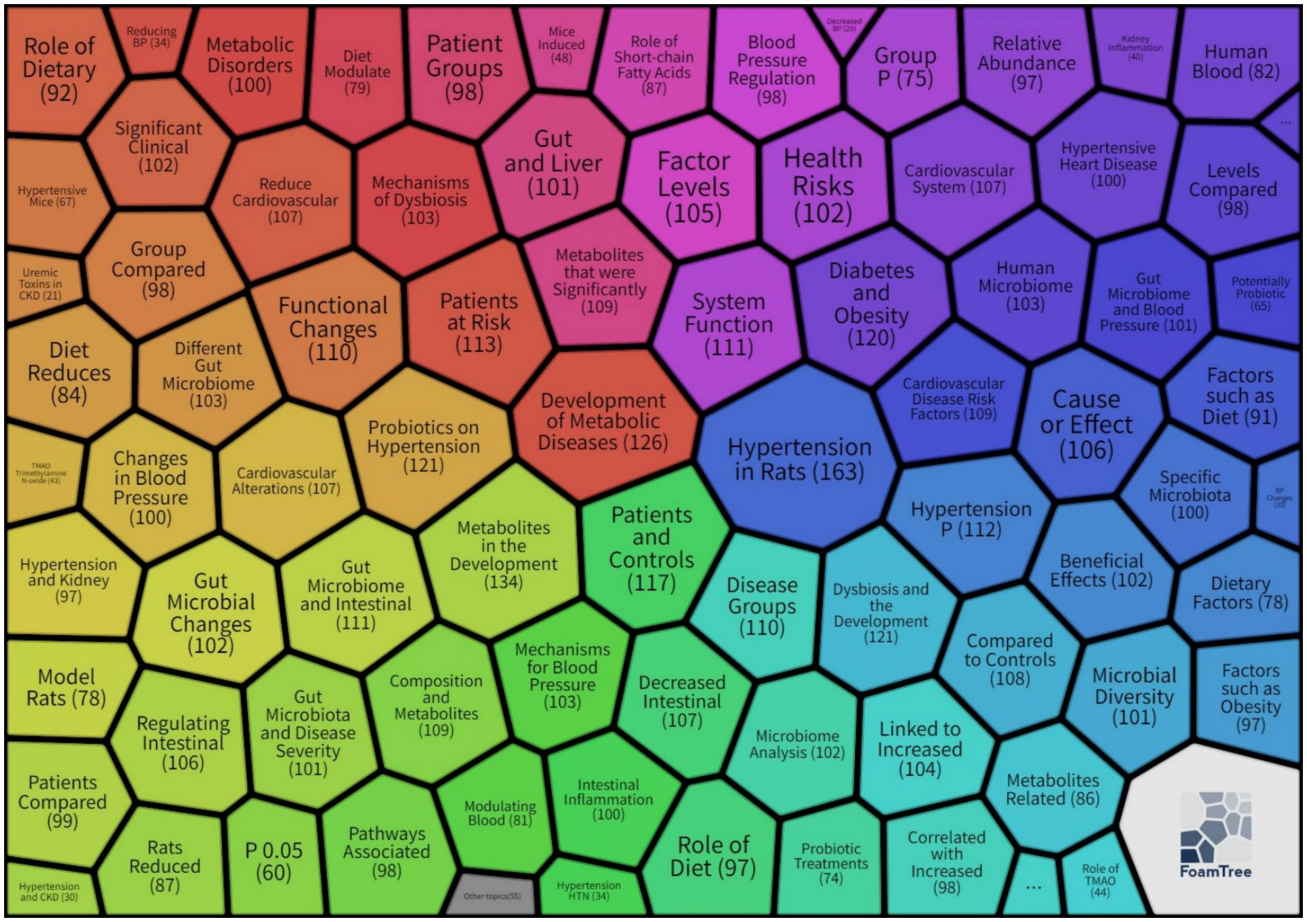
Keyword clustering hotspot map for hypertension and gut microbiota research.

Traditional review methods inadequately capture the dynamic evolution of complex knowledge networks. This study employed FoamTree visualization (Figure 1) to generate a “research hotspot distribution map” for hypertension and gut microbiota keyword clustering. This tool transforms complex keyword co-occurrence networks into thematic modules using hexagonal nesting with color-coded zones, where colors distinguish research clusters, hexagon sizes correspond to keyword frequencies, and nested structures reveal hierarchical relationships from broad themes to specific research directions. The visualization encompasses six core clustering modules: red clusters examine metabolic disorders and disease risk, investigating how microbial dysbiosis triggers hypertension through metabolic dysfunction; purple clusters address cardiovascular systems and health risks, emphasizing the gut-heart axis and microbial characteristics in patients with diabetes and obesity comorbidities; green clusters investigate underlying mechanisms of microbial blood pressure regulation, including inflammatory pathways and related metabolite effects; blue clusters explore microbial characteristics in hypertensive patients and intervention efficacy; yellow/orange clusters focus on microbiota-blood pressure associations and intervention validation in animal models; cyan clusters study dietary influences on microbiota and blood pressure through nutritional intervention pathways. However, visualization alone provides insufficient understanding of the hypertension and gut microbiota research landscape. Therefore, we employed bibliometric analysis to quantitatively examine citation relationships, collaboration patterns, and thematic associations, achieving a three-dimensional perspective of field development: structurally, identifying collaboration networks among key countries, institutions, and authors; temporally, revealing evolutionary trajectories of research hotspots, such as progression from early correlation analyses toward causal mechanism validation; and contextually, clustering knowledge foundations and identifying frontier topics, including emerging concepts like the microbiota-brain-gut axis.

To address methodological limitations in existing reviews, this study integrates the complementary strengths of CiteSpace and VOSviewer analytical tools—CiteSpace excels in temporal evolution analysis and burst detection, while VOSviewer specializes in collaboration network construction and co-occurrence clustering. Based on Web of Science Core Collection data from 2000 to 2025, this study aims to: construct collaboration network maps analyzing cooperation intensity and pivotal roles among countries, institutions, and authors; identify knowledge foundations and evolutionary pathways by combining co-citation analysis with temporal zone visualization to establish a three-phase developmental model; and detect research frontiers through keyword burst analysis to identify potential future directions, such as artificial intelligence-driven precision microbiota interventions. These findings will provide valuable references for future researchers and facilitate comprehensive understanding of the current field landscape.

## 2 Methods

### 2.1 Data sources and search strategy

Data were retrieved from the Web of Science Core Collection (WoSCC) using Boolean logic to construct a comprehensive search strategy: TS = (“hypertension” OR “high blood pressure” OR “hypertensive” OR “hyperpiesis” OR “hypertonia” OR “renovascular hypertensive” OR “essential hypertension” OR “primary hypertension” OR “genetic hypertension” OR “resistant hypertension” OR “secondary hypertension” OR “renal parenchymal hypertension”) AND TS = (((“gut” OR “gastrointestin*” OR “intestin*” OR “gastro-intestin*”) AND (“microbiot*” OR “flora” OR “bacteria” OR “microflora” OR “microbiome*”)) OR “probiotic” OR “prebiotic” OR “antibiotic” OR “dysbiosis”).

**Figure 2:**
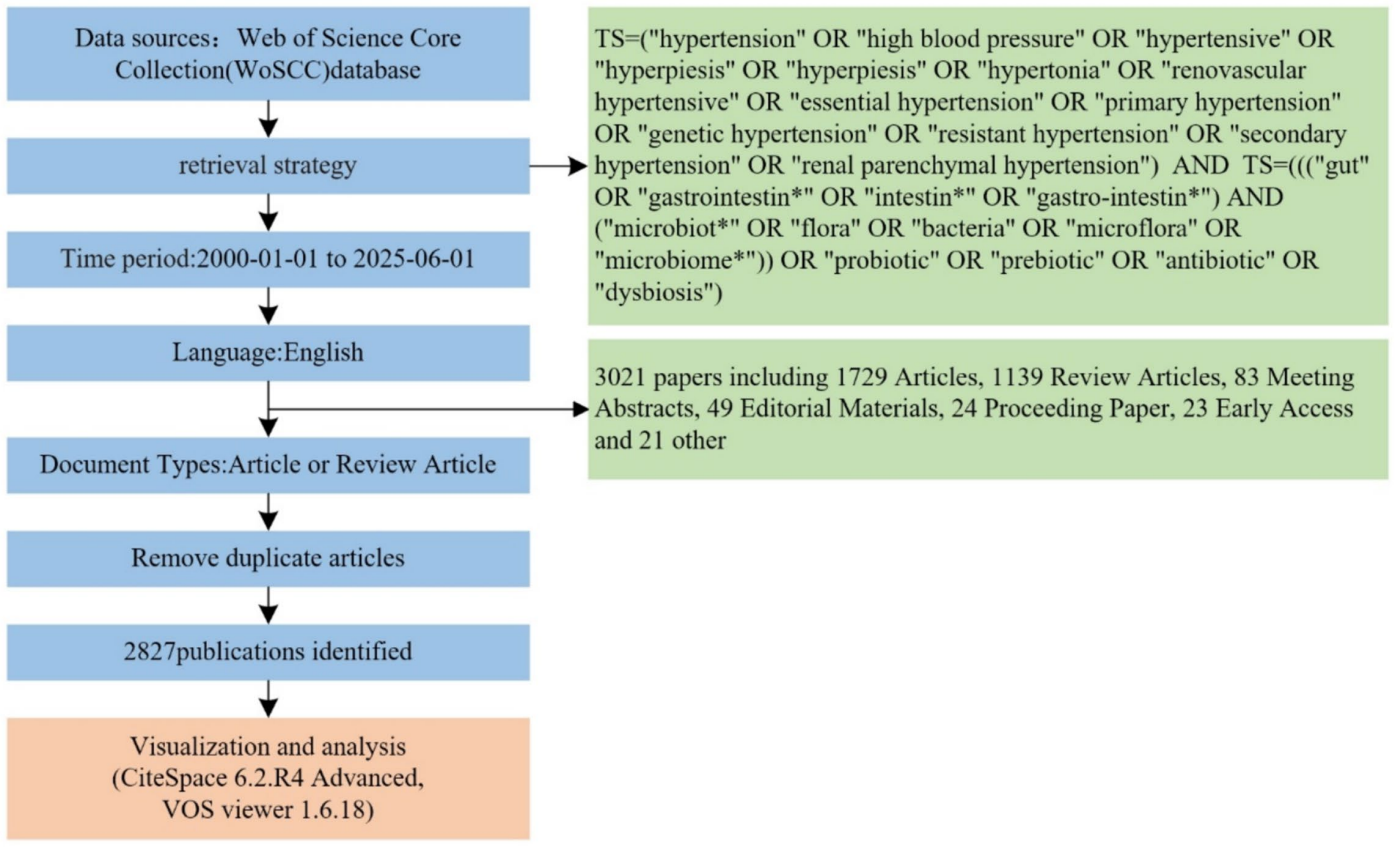
Flowchart of data screening and bibliometric analysis process.

The search timeframe spanned from January 1, 2000, to June 1, 2025, with document types restricted to English-language research articles and review articles. Initial screening yielded 3,021 publications. After removing duplicates and excluding non-research documents such as conference abstracts and editorials (195 documents), 2,827 valid publications were included for analysis. Visualization analysis was conducted using CiteSpace 6.2. R4 and VOSviewer 1.6.18, with data processing procedures including institutional name standardization and author name variant consolidation, as illustrated in Figure 2.

### 2.2 Visualization analysis tools

This study employed CiteSpace (version 6.2. R4) and VOSviewer (version 1.6.18) for co-occurrence analysis and visualization mapping, with analytical results subsequently imported into Microsoft Excel 2019 for chart generation. In visualization maps, nodes and connecting lines form the primary elements, with each node representing an analyzed unit such as authors, institutions, or journals. Connecting lines facilitate understanding of co-occurrence and co-citation relationships between nodes. Generally, larger and more frequent nodes indicate higher occurrence rates, while the quantity and thickness of connecting lines between nodes reflect relationship strength. Centrality serves as a metric for measuring node importance within networks. Nodes with higher numbers of shortest paths passing through them exhibit stronger centrality. When a node’s centrality value exceeds 0.1, it is typically considered a pivotal point within the research domain.

## 3 Results

### 3.1 Annual publication volume statistics and analysis

**Figure 3:**
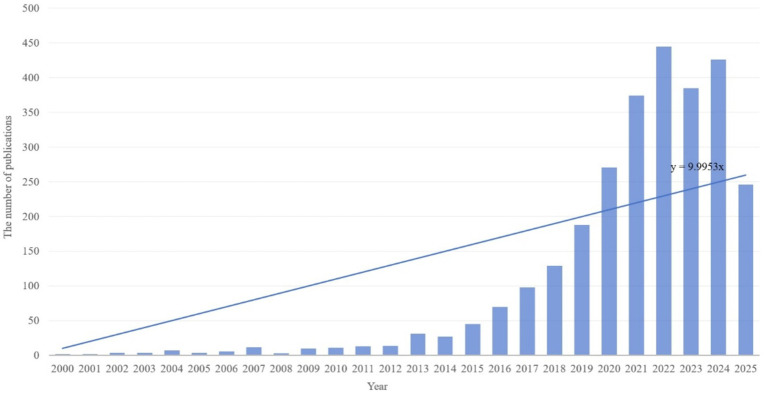
Global annual publication trends in hypertension and gut microbiota research (2000–2025).

This study analyzed publication volumes in hypertension and gut microbiota research from 2000 to 2025 (Figure 3). The overall trend demonstrates sustained growth in research output throughout this period, with linear regression analysis revealing an annual increase rate of approximately y = 9.9953x publications per year. Based on research intensity evolution patterns, the development can be categorized into three distinct phases: slow accumulation period (2000–2013), accelerated growth period (2014–2019), and high-level stabilization period (2020–2025). The slow accumulation period (2000–2013) was characterized by annual publication volumes consistently below 50 articles. Technical limitations and research paradigm constraints restricted interdisciplinary investigation to preliminary exploration, accumulating only limited phenomenological evidence. The accelerated growth period (2014–2019) witnessed breakthrough advances in multi-omics technologies and landmark theoretical studies, driving publication volumes from approximately 30 to nearly 200 articles annually. Research focus shifted from phenomenological description toward mechanistic investigation and clinical exploration, establishing an interdisciplinary research ecosystem. The high-level stabilization period (2020–2025) maintained publication volumes between 250 and 450 articles annually. The field transitioned from “quantitative expansion” to “rational deepening,” focusing on innovative directions and clinical challenges while sustaining high research activity through strategic adjustments. Overall, the field demonstrates a clear long-term growth trajectory driven by technological advances, clinical demand, and interdisciplinary collaboration. Future developments in precision interventions, multi-omics mechanisms, and global cohort studies are expected to sustain publication innovation and facilitate clinical translation progress.

**Figure 4:**
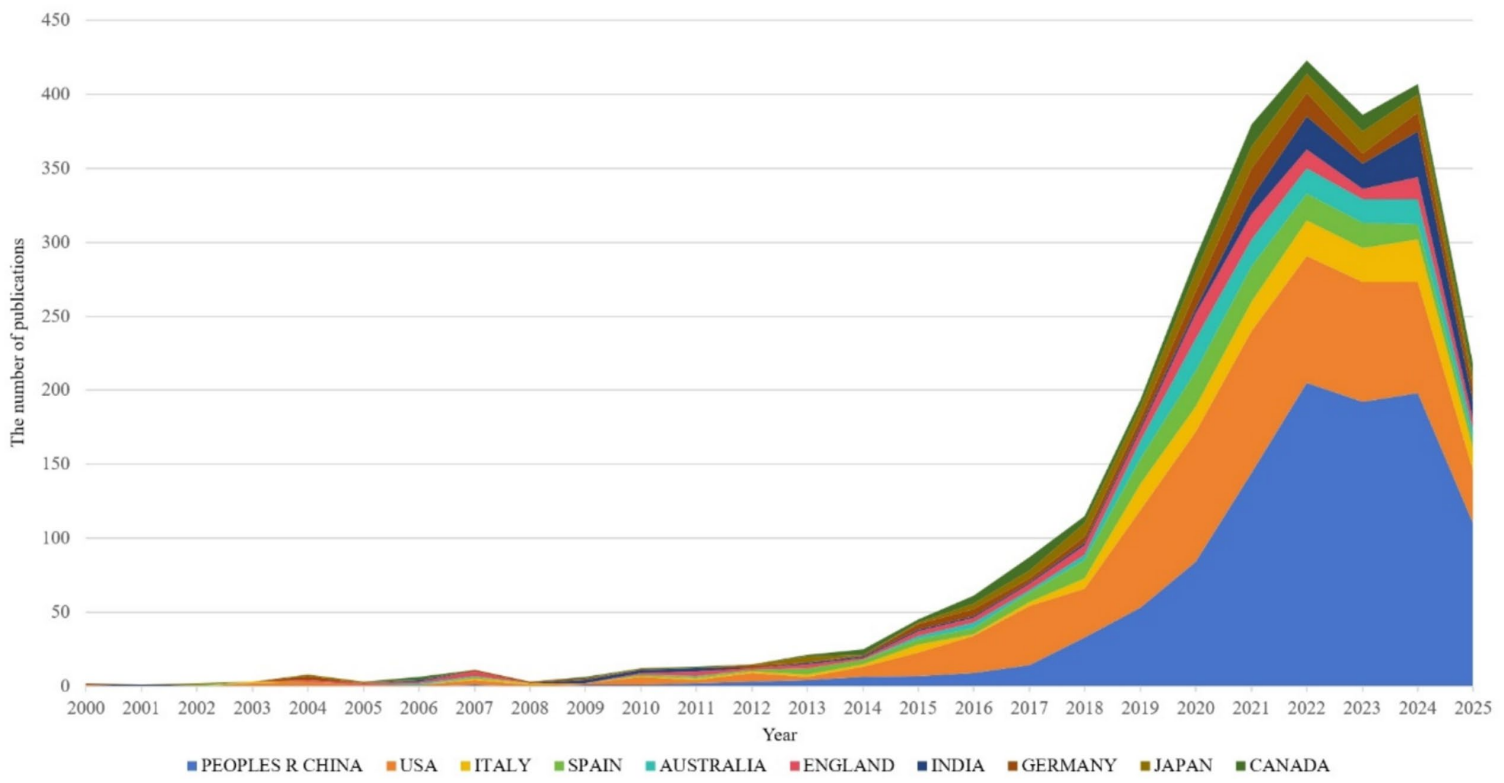
Distribution of cumulative publication proportions by major countries.

Dynamic changes in publication output from top 10 countries in hypertension and gut microbiota research (2000–2025), during 2000–2013, all countries maintained extremely low publication volumes, reflecting limited global attention to this research field in its early stages. After 2014, coinciding with rising research interest, publication volumes from all countries accelerated rapidly, peaking during 2020–2023 with over 400 publications annually. China (blue) and the United States (orange) emerged as primary contributors, with China’s publication share continuously expanding over time, becoming the leading contributor after 2022, demonstrating the rise of domestic research capabilities. The United States maintained consistently high output, reflecting its established expertise in this research domain. Although Italy, Spain, and other countries contributed relatively fewer publications, they collectively formed a global research network, demonstrating a collaborative pattern characterized by “China-US leadership with multinational participation” (Figure 4)

This distribution is driven by national research resources and hypertension disease burden, exemplified by China’s large hypertensive population and urgent clinical needs. It also relates to international collaboration networks, with China and the US leading multicenter studies that establish foundations for global collaborative innovation, while highlighting the need for increased research investment in developing countries (such as India) to address gaps in population heterogeneity studies.

### 3.2 Bibliometric analysis of countries, institutions, and authors

**Figure 5:**
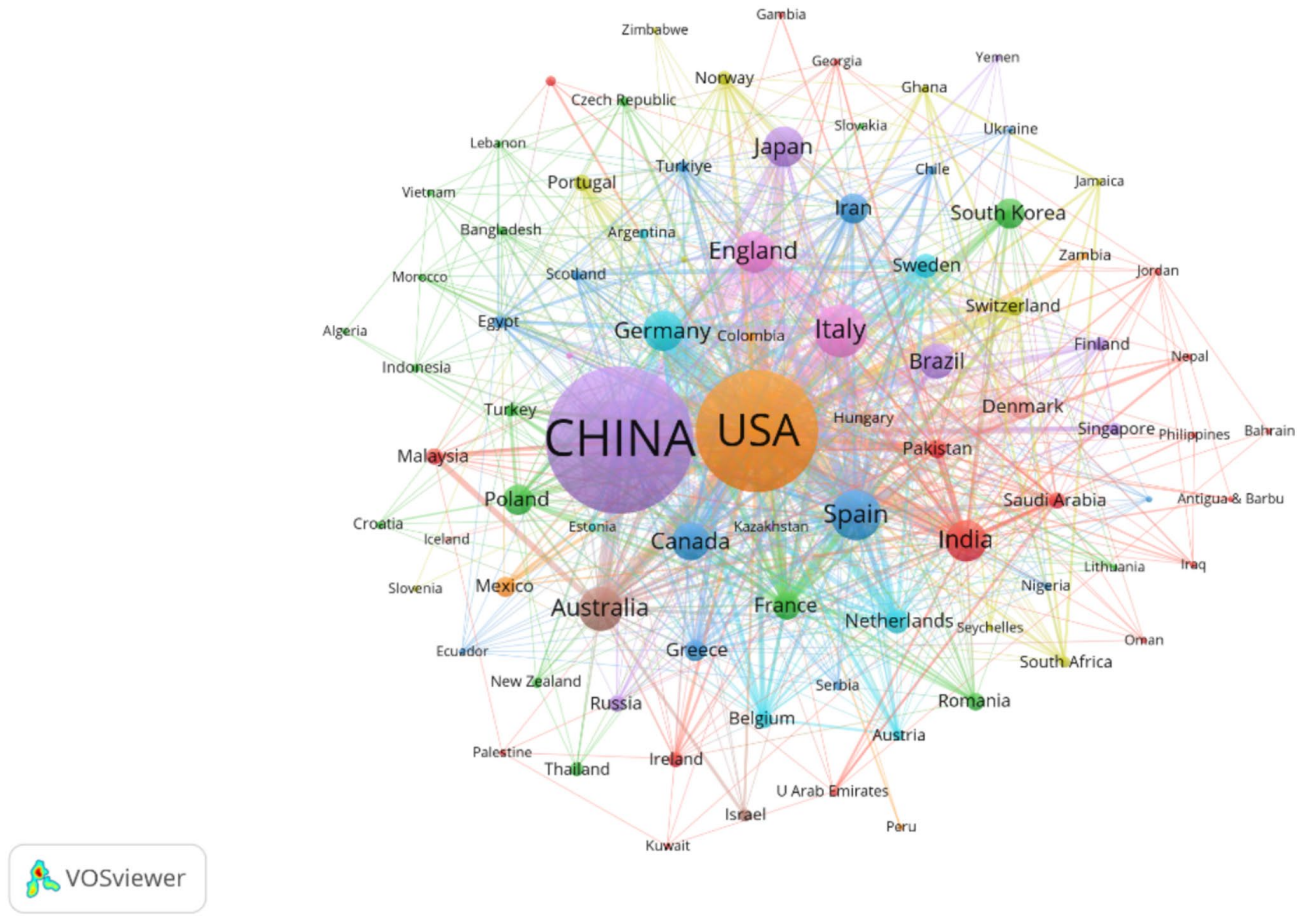
Presents the national collaboration network, where node size represents publication volume, line thickness indicates collaboration frequency, and node color denotes cluster membership. The figure includes 86 countries, each with at least two publications. The network reveals ten distinct collaborative clusters, with an overall connection strength of 1,877.

**Figure 6:**
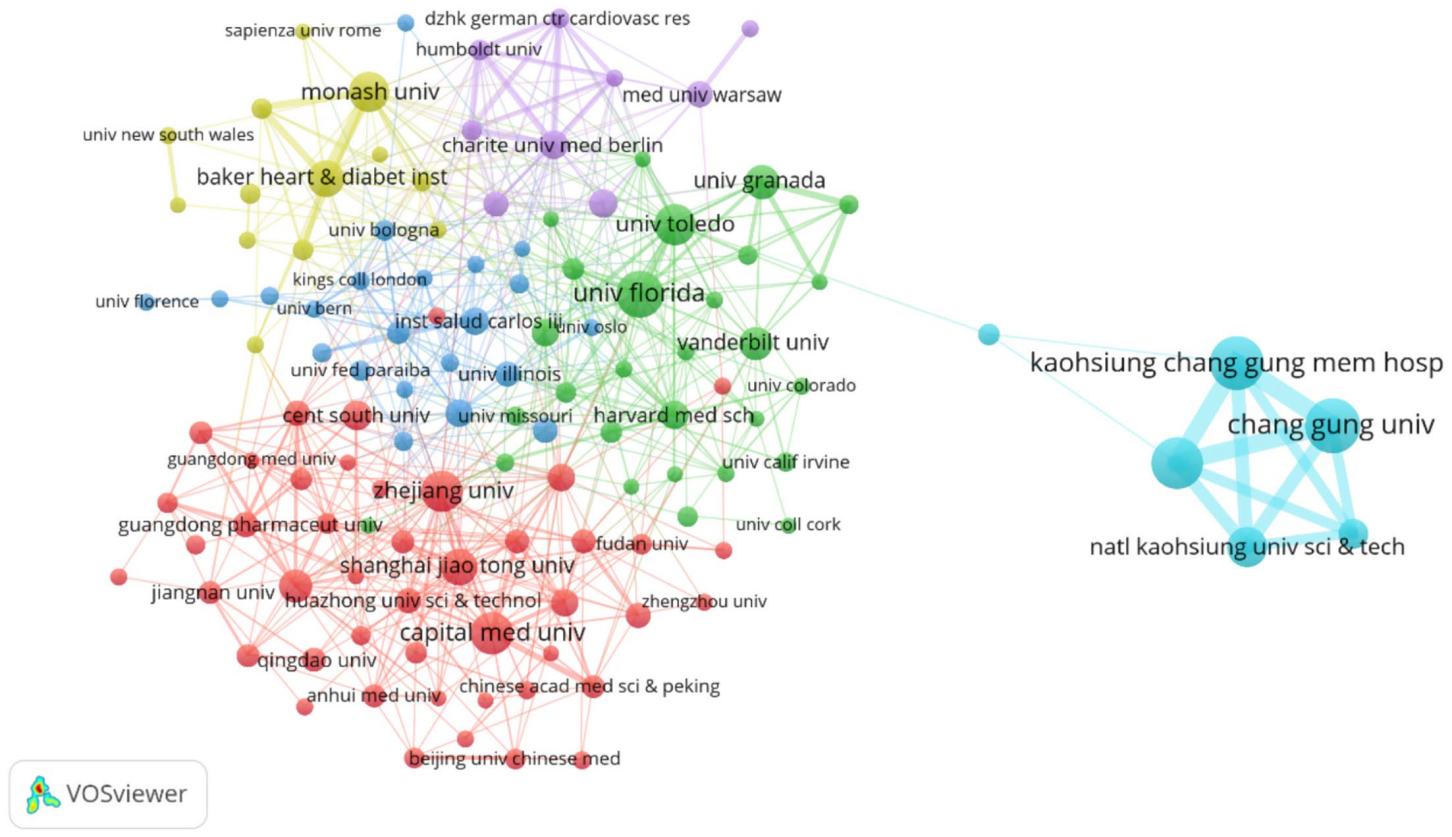
Illustrates the institutional collaboration network, where node size corresponds to the number of publications, line thickness reflects the frequency of collaboration, and node color represents different collaborative groups. Among the 3,836 institutions analyzed, the figure highlights 123 institutions with at least 10 publications each. The network is organized into six distinct collaborative clusters, with a total connection strength of 1,537.

**Figure 7:**
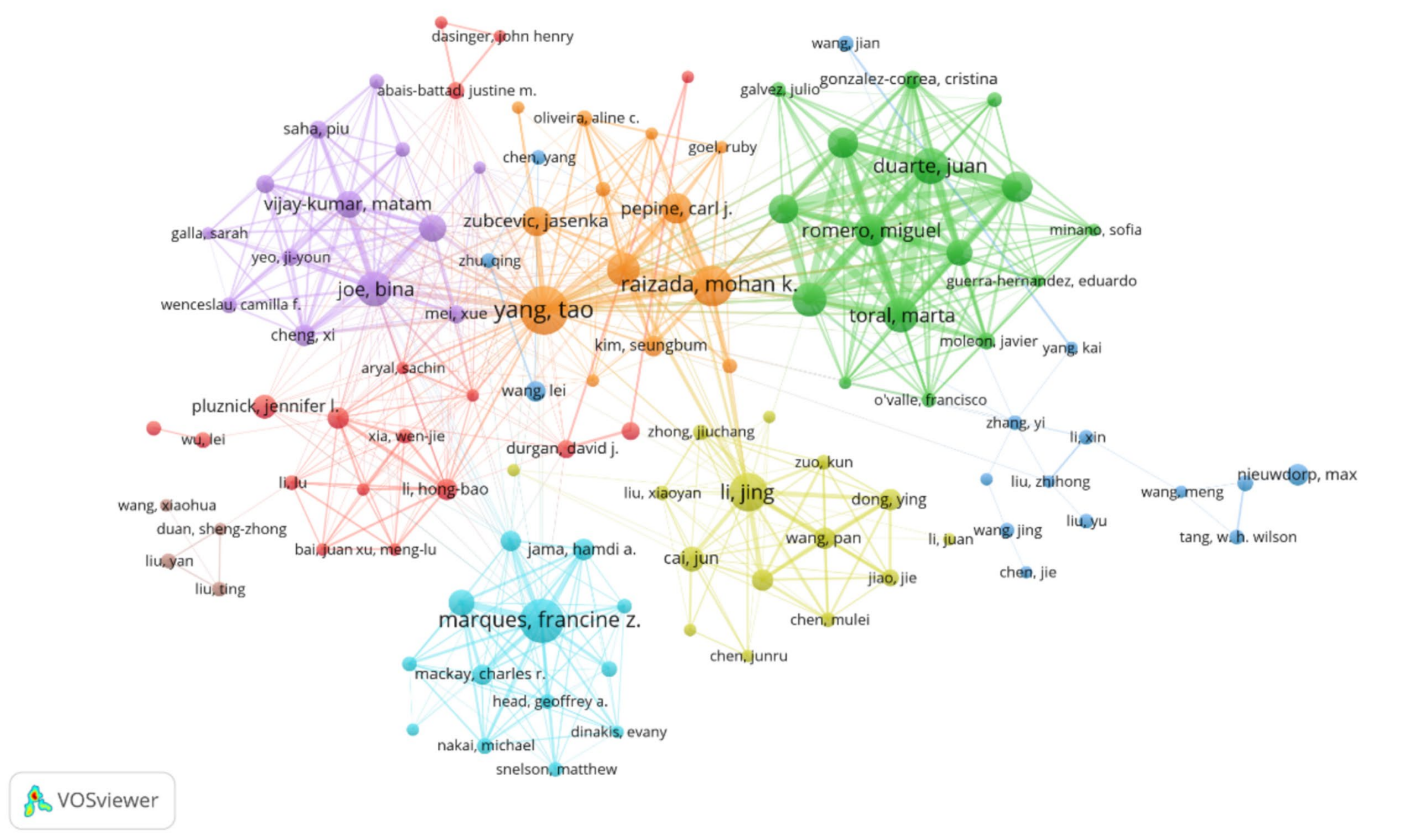
Depicts the author collaboration network, where node size indicates the number of publications, line thickness represents collaboration frequency, and node color distinguishes collaborative groups. The visualization includes 108 authors with at least five publications each. These authors are organized into eight distinct collaborative clusters, with a total network connection strength of 2,576.

This study employs VOSviewer to visualize country collaboration networks, institutional cooperation networks, and author collaboration networks (Figures 5–7), combined with bibliometric data (Tables 1–4), to systematically analyze the global distribution, collaborative patterns, and key contributors in hypertension and gut microbiota research (van Eck and Waltman, 2010). Results reveal an international collaboration pattern characterized by China-US leadership with global coordination, where leading institutions and author teams play dominant roles.

**Table 1:** Lists the top 10 most productive countries, detailing the number of publications, total citations, number of collaborative links, and overall collaboration strength for each country or region.

Rank	Country	Documents	Citations	Links	TLS
1	China	918	3,671	50	162
2	United States	676	64	71	287
3	Italy	171	64	41	94
4	Spain	154	3,555	39	81
5	Australia	124	85	37	61
6	England	114	254	51	109
7	India	113	748	43	40
8	Germany	110	6,688	46	83
9	Japan	110	224	24	29
10	Canada	97	27	41	72

**Table 2:** Presents the top 10 most productive institutions, showing the number of publications, total citations, number of collaborative links, and overall collaboration strength for each institution.

Rank	Organization	Documents	Citations	Links	TLS
1	Chang Gung University	78	38	6	108
2	Kaohsiung Medical University	69	9	8	104
3	University of Florida	59	33	35	18
4	Capital Medical University	51	154	32	5
5	Zhejiang University	48	54	49	9
6	University of Toledo	47	19	44	18
7	National Kaohsiung University of Science and Technology	46	41	6	82
8	Monash University	45	8	39	38
9	Baker Heart and Diabetes Institute	40	15	37	39
10	Shanghai Jiao Tong University	39	11	27	5

**Table 3:** Top 10 most productive authors.

Rank	Author	Documents	Citations	TLS	Affiliated institution
1	Tain, You-Lin	74	316	137	Chang Gung University, China
2	Hsu, Chien-Ning	65	1,627	133	Kaohsiung Medical University, China
3	Hou, Chih-Yao	44	78	110	National Kaohsiung University of Science and Technology, China
4	Yang, Tao	44	361	33	University of Florida, United States
5	Marques, Francine Z.	37	176	1	Baker Heart and Diabetes Institute, Australia
6	Raizada, Mohan K.	33	131	37	University of Florida, United States
7	Chang-Chien, Guo-Ping	29	99	86	Cheng Shiu University, China
8	Jing Li	29	123	11	Capital Medical University, China
9	Duarte, Juan	27	176	35	University of Granada, Spain
10	Toral, Marta	25	552	35	Centro Nacional de Investigaciones Cardiovasculares, Spain

**Table 4:** Top 10 most frequently co-cited authors.

Rank	Author	Co-citations	Affiliated institution
1	Tao Yang	664	University of Florida, United States
2	Jing Li	628	Capital Medical University, China
3	Tang, W. H. Wilson	475	Cleveland Clinic, United States
4	Pluznick, Jennifer L.	424	The Johns Hopkins School of Medicine, United States
5	Marques, Francine Z.	412	Baker Heart and Diabetes Institute, Australia
6	Cani, Patrice D	367	Catholic University of Louvain, Belgium
7	Kim, Seungbum	346	University of Florida, United States
8	Peter J. Turnbaugh	339	Harvard University, United States
9	Wang, Zeneng	339	Cleveland Clinic, United States
10	Santisteban, Monica M.	285	Vanderbilt University School of Medicine, United States

The country collaboration network (Figure 5) reveals a core-periphery structure led by China and the US. The US maintains strong collaborations with Germany (Links = 46) and Spain (Links = 39), focusing on microbiota metabolic mechanisms. China collaborates closely with Japan (Links = 24) and South Korea (Links = 18), emphasizing population cohorts and clinical translation. Notably, developing countries like India (113 publications, Links = 43) and Brazil (89 publications, not ranked) are gradually integrating into the network, though their publication output represents only 12.3% of China’s, reflecting uneven global research resource distribution.

Table 1 demonstrates that China (918 publications) and the US (676 publications) form the field’s dual engines, with publication volumes far exceeding Italy (171 publications) and Spain (154 publications). China leverages its large hypertensive patient population (over 300 million cases) and policy support such as the Precision Medicine Initiative to achieve significant advantages in population cohort studies. The US maintains a leading position in microbiota metabolic mechanism analysis through accumulated foundational microbiology research. Regarding research impact, Germany (Citations = 6,688) and Spain (Citations = 3,555) demonstrate superior research quality despite lower publication volumes than China and the US, with higher proportions of highly cited papers (Germany 12.3%, Spain 11.7%) compared to China (5.8%) and the US (9.2%).

The institutional collaboration network (Figure 6) exhibits regional clustering with cross-domain coordination characteristics. Chinese universities (such as Zhejiang University and Shanghai Jiao Tong University) form a Yangtze River Delta collaboration circle focusing on population-specific microbiota mechanisms. US universities (including University of Florida and Vanderbilt University) establish a southeastern collaboration network emphasizing metabolomics and causal inference. Emerging institutions like University of São Paulo in Brazil and Indian Institute of Technology integrate into the network through international collaboration, though their collaboration intensity with core institutions (TLS < 20) remains significantly lower than Chinese and US universities.

Table 2 reveals that Taiwan institutions lead the rankings, with Chang Gung University (78 publications) and Kaohsiung Medical University (69 publications) occupying the top two positions. Their advantages stem from Taiwan’s specialized funding for precision medicine and microbiota regulation, such as Chang Gung University’s Center for Gut Microbiota and Metabolic Diseases Research, focusing on probiotic interventions and clinical translation. University of Florida (59 publications) and Capital Medical University (51 publications) follow among Chinese and US institutions. University of Florida leverages its Microbiome and Cardiovascular Disease Laboratory to consistently produce highly cited research on short-chain fatty acid receptor signaling pathways, while Capital Medical University utilizes Beijing’s top-tier hospital resources to conduct multicenter studies on gut microbiota and hypertension complications.

The author collaboration network (Figure 7) demonstrates a multi-center aggregation pattern. Teams led by Raizada, Mohan K. (University of Florida) and Yang, Tao (University of Florida) form a southeastern US core circle focusing on microbiota metabolic mechanisms and causal validation. Teams led by Tain, You-Lin (Chang Gung University) and Hsu, Chien-Ning (Kaohsiung Medical University) establish a Taiwan collaboration network emphasizing clinical translation and probiotic interventions. Notably, cross-regional collaborative teams (such as Yang, Tao’s collaboration with Zhejiang University) represent only 15% of partnerships, reflecting the geographical clustering of core research teams.

Table 3 shows Taiwan authors dominating the high-productivity rankings, with Tain, You-Lin (74 publications) and Hsu, Chien-Ning (65 publications) leading the list. Their research focuses on probiotic interventions for hypertension, with Tain’s team demonstrating that *Lactobacillus plantarum* can reduce blood pressure by regulating intestinal barrier function. Table 4 indicates that Chinese and US authors, including Tao Yang (664 citations) and Jing Li (628 citations), dominate the high-impact rankings. Tao Yang’s team published research on the short-chain fatty acid-gut-vascular axis in Circulation Research, establishing a molecular model for microbiota metabolite regulation of blood pressure (Yang et al., 2015). Jing Li′s team used Mendelian randomization to validate the causal relationship between reduced microbiota diversity and increased hypertension risk (He et al., 2023).

In summary, the author community in hypertension and gut microbiota research exhibits regional clustering leadership with insufficient cross-regional collaboration. Taiwan has emerged as a highly productive region due to its clinical resources and international collaboration traditions. Future efforts should focus on improving international cooperation mechanisms, establishing collaborative innovation platforms, and consolidating global research efforts to address core challenges such as mechanism-to-application translation, ultimately providing more universally applicable gut microbiota intervention strategies for hypertension prevention and control.

### 3.3 Bibliometric analysis of keyword co-occurrence and clustering

**Figure 8:**
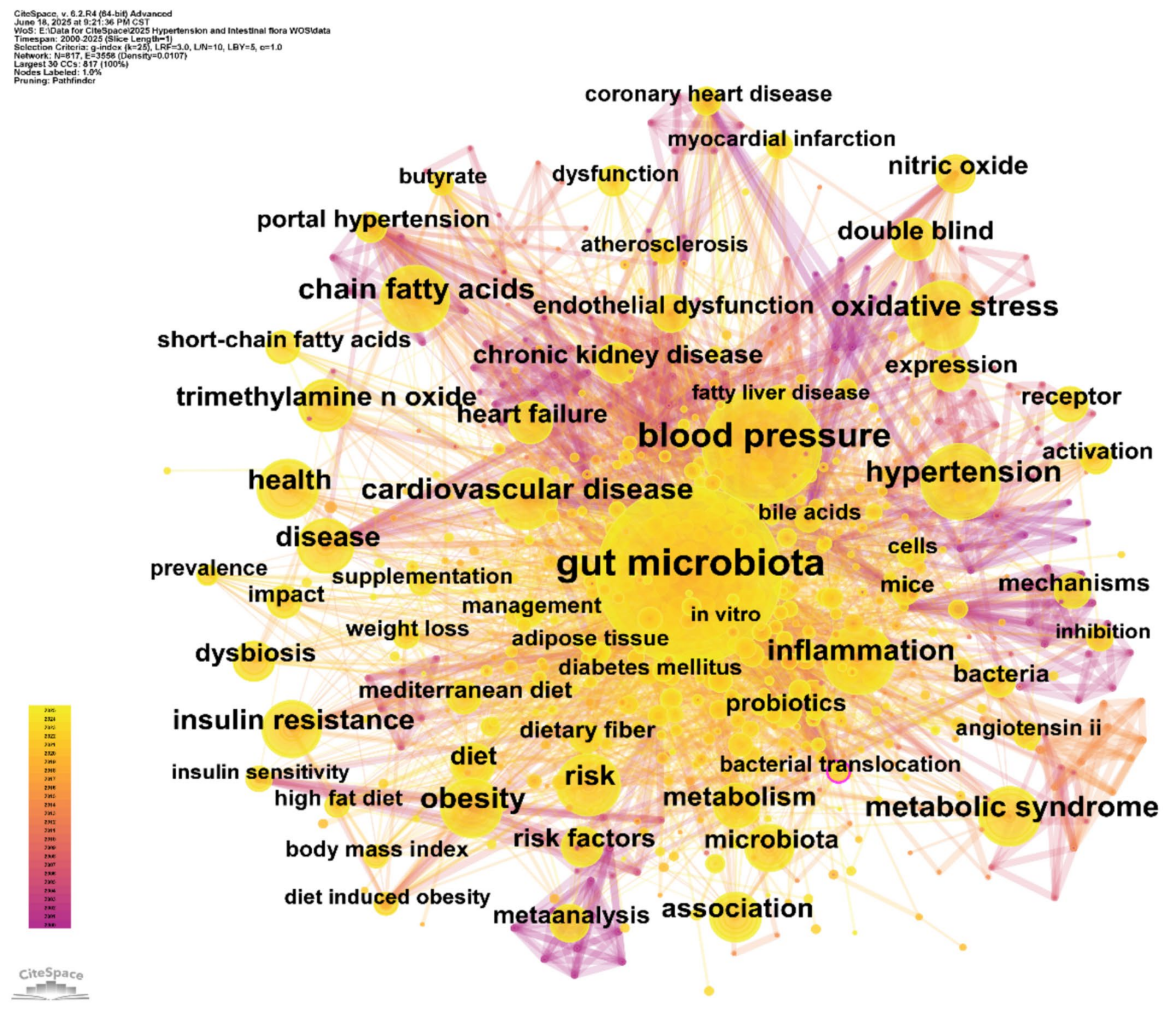
Keyword co-occurrence network. Each node represents a keyword; node size reflects the number of articles in which the keyword appears, and the links indicate associations between keywords.

Keyword co-occurrence refers to the phenomenon where different keywords appear together within the same publication. Analyzing the frequency and network structure of co-occurring keywords helps reveal the intrinsic connections among research topics allowing for the precise identification of key hotspots and the underlying knowledge structure within the field. Frequently co-occurring keyword pairs often indicate major research directions while keywords with high centrality act as “bridges” connecting different topics facilitating interdisciplinary collaboration and knowledge integration (Chen, 2017). Using the keyword co-occurrence network generated by CiteSpace (Figure 8) and the associated data table (Table 5) we systematically examined research hotspots thematic associations and evolving trends in the field of hypertension and gut microbiota thereby delineating the core structure of the discipline.

**Table 5:** Top 10 keywords by frequency and centrality.

Rank	Keywords	Frequency	Keywords	Centrality
1	Gut microbiota	1,720	Blood pressure	0.07
2	Blood pressure	781	Portal hypertension	0.07
3	Hypertension	365	Bile acids	0.06
4	Oxidative stress	296	Hypertension	0.05
5	Inflammation	296	Disease	0.04
6	Cardiovascular disease	290	Double blind	0.04
7	Chain fatty acids	274	Microbiota	0.04
8	Metabolic syndrome	251	Nitric oxide	0.04
9	Obesity	246	Mice	0.04
10	Insulin resistance	243	Coronary heart disease	0.04

In the keyword co-occurrence network (Figure 8), node size is proportional to keyword frequency, while the edges represent the strength of thematic connections. The dense interconnections among keywords reflect the complex relationships within the field. “Gut microbiota” (frequency: 1,720) and “blood pressure” (frequency: 781) are at the core of the network, directly linked to other key nodes such as “hypertension” (365) and “cardiovascular disease” (290). This highlights the central logic of the field, which focuses on the “gut microbiota–blood pressure regulation–cardiovascular disease” pathway. The frequent co-occurrence of these terms underscores the prominence of gut microbiota and blood pressure regulation as primary research themes.

By combining frequency and centrality data, keywords such as “oxidative stress” (frequency: 296, centrality: 0.04) and “inflammation” (frequency: 296, centrality: 0.04) emerge as crucial bridges connecting gut microbiota and hypertension. This highlights the fundamental role of oxidative stress and inflammatory pathways in microbiota-mediated blood pressure regulation. Similarly, clusters of metabolic terms such as “chain fatty acids” (274) and “trimethylamine N-oxide (TMAO)” (a high-frequency node) emphasize the research interest in microbial metabolites and their disease associations. This aligns with clinical evidence linking elevated TMAO to atherosclerosis and increased blood pressure (Huc et al., 2018; Qi et al., 2018). Frequently occurring keywords like “hypertension” and “cardiovascular disease” remain at the center of clinical focus, while keywords with high centrality, such as “blood pressure” (centrality: 0.07), serve as pivotal connectors across multiple research themes, supporting the multidimensional “microbiota–metabolism–disease” framework.

The network topology reveals three major thematic clusters: (1) The “gut microbiota–metabolic regulation” cluster centers on “gut microbiota” and connects to keywords like “chain fatty acids,” “bile acids,” and “metabolic syndrome.” This reflects research evolving from characterizing microbiota composition to elucidating the metabolic functions of microbial products (e.g., short-chain fatty acids and bile acids) in host metabolism and blood pressure regulation. (2) The “hypertension–cardiovascular comorbidities” cluster revolves around “blood pressure” and “hypertension,” linking to terms such as “coronary heart disease” and “chronic kidney disease.” This underscores clinical research on the associations between gut microbiota and hypertension-related complications, such as reduced microbial diversity and microbial translocation exacerbating systemic inflammation and blood pressure abnormalities in kidney disease patients (Liu et al., 2022; Ren et al., 2024). (3) The “intervention strategies and clinical research” cluster includes keywords like “probiotics,” “diet,” and “double-blind,” corresponding to clinical investigations of probiotic supplementation and Mediterranean diet interventions. These terms echo the transition from mechanistic studies to translational research and are aligned with keywords such as “supplementation” and “management” in the network.

In summary, the keyword co-occurrence analysis reveals the establishment of a core research framework in the field of hypertension and gut microbiota, characterized by a “microbiota–metabolism–disease” chain. Oxidative stress and inflammatory pathways are highlighted as key mechanistic foci, while probiotics and dietary interventions represent major directions for translational research.

By applying keyword clustering analysis using CiteSpace to literature on hypertension and gut microbiota, we can systematically elucidate the thematic structure and internal relationships within the field. The algorithm groups semantically related keywords into thematic clusters, each representing a distinct research direction. Strong intra-cluster associations reflect concentrated research foci, while inter-cluster connections indicate interdisciplinary trends, providing a structured perspective for understanding the knowledge landscape. Key parameters for cluster interpretation include: Silhouette score [quantifying cluster cohesion and separation, ranging from 0 to 1; values >0.5 indicate effective clustering, and >0.7 suggest highly robust clusters, reflecting strong internal consistency and clear differentiation between themes (Bao et al., 2019)]; Mean Year (indicating the period of research activity for each cluster, with higher mean years signifying emerging or active research areas); and Label (core thematic terms extracted using Latent Semantic Indexing, summarizing the research focus of each cluster and simplifying network interpretation).

For this study, the clustering analysis was configured as follows: the time span covered 2000–2025, with annual time slices (Slice Length = 1) to accurately capture the temporal evolution of research topics. The node type was restricted to “Keywords” to focus on central concepts and minimize noise from non-essential nodes. The Pathfinder algorithm was employed to prune the network, removing redundant weak links and highlighting the core topological structure—thereby improving the interpretability and focus of the clusters. In terms of cluster quality and validity, the results were robust: the overall silhouette score was 0.7276, and all 10 sub-clusters had silhouette values above 0.5 (ranging from 0.564 to 0.942)—for example, cluster #8 (lactic acid bacteria, 0.942) and cluster #9 (blood pressure, 0.904) were particularly cohesive. The modularity Q index reached 0.4902, well above the conventional threshold of 0.3, confirming the statistical significance of the clustering and the substantive alignment of themes with research practice.

**Figure 9:**
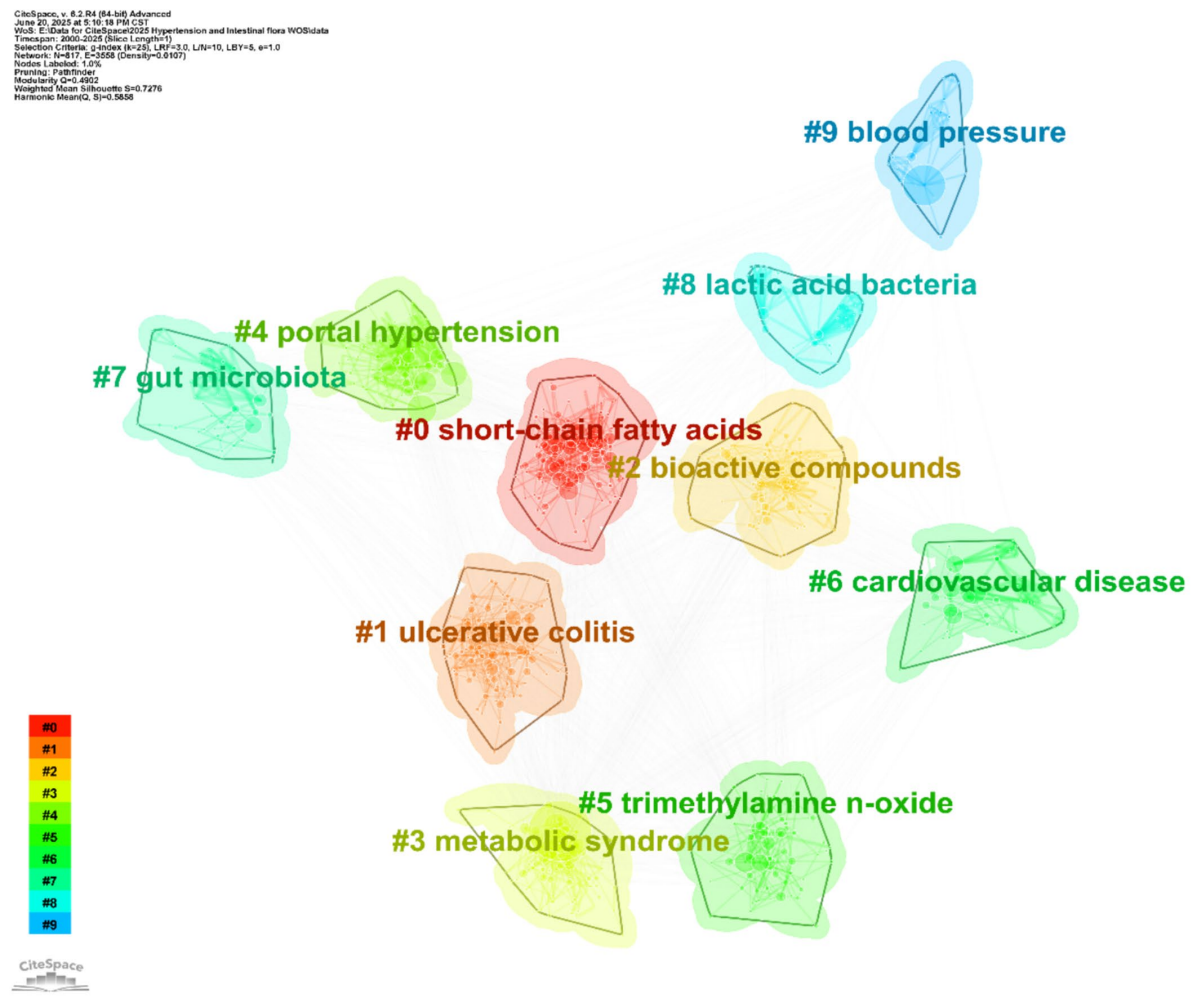
Keyword clustering map. The map reveals 10 distinct thematic clusters, each represented by a different color: #0 short-chain fatty acids, #1 ulcerative colitis, #2 bioactive compounds, #3 metabolic syndrome, #4 portal hypertension, #5 trimethylamine n-oxide, #6 cardiovascular disease, #7 gut microbiota, #8 lactic acid bacteria, #9 blood pressure.

Cluster analysis identified 10 distinct thematic clusters (see Table 6 and Figure 9), each representing a core research dimension in the field. Cluster #0 (“short-chain fatty acids”; silhouette 0.626, mean year 2017) focuses on the relationship between short-chain fatty acids and gut microbiota, including topics such as isolated systolic/diastolic hypertension and microbial metabolites—highlighting the role of short-chain fatty acids in blood pressure regulation and urinary system disorders. Cluster #1 (“ulcerative colitis”; silhouette 0.564, mean year 2019) centers on gut microbiota and oxidative stress, linking to non-alcoholic fatty liver disease and fecal microbiota transplantation, and reflects the interplay of gut diseases, microbiota, metabolism, and vascular stiffness. Cluster #2 (“bioactive compounds”; silhouette 0.612, mean year 2017) involves short-chain and bioactive compounds, probiotics, and cardiovascular disease, emphasizing research on microbiota-derived bioactives, dietary patterns, and obesity management. Cluster #3 (“metabolic syndrome”; silhouette 0.686, mean year 2016) focuses on gut microbiota and metabolic syndrome, encompassing fatty acids, insulin resistance, and animal models, and reflects research on metabolic disorders linked to blood pressure. Cluster #4 (“portal hypertension”; silhouette 0.811, mean year 2008) investigates gut microbiota and portal hypertension, including sleep apnea and intestinal permeability, and highlights mechanisms linking the microbiota, liver injury, and disease progression. Cluster #5 (“trimethylamine N-oxide”; silhouette 0.64, mean year 2018) addresses the association of TMAO with chronic kidney disease and cardiovascular disease, reflecting the role of this metabolite in blood pressure regulation and hepatorenal disorders. Cluster #6 (“cardiovascular disease”; silhouette 0.815, mean year 2011) explores the connection between cardiovascular disease and gut microbiota, including precision nutrition, dietary interventions, and histone acetylation, underlining the interplay of disease prevention/treatment, microbiota, and nutritional strategies. Cluster #7 (“gut microbiota”; silhouette 0.816, mean year 2011) is centered on gut microbiota, covering bacterial translocation, immune tolerance, and network pharmacology, reflecting fundamental mechanisms and associations with multiple diseases. Cluster #8 (“lactic acid bacteria”; silhouette 0.942, mean year 2005) focuses on lactic acid bacteria and fermented foods, including metabolic syndrome and oxidative stress, reflecting research on probiotics and microbiota modulation. Cluster #9 (“blood pressure”; silhouette 0.904, mean year 2010) is devoted to blood pressure and gut microbiota, linking to sleep apnea, TMAO, and endothelial dysfunction, and highlights the role of the microbiota in blood pressure regulation and related pathologies.

**Table 6:** Keyword cluster analysis.

Cluster ID	Silhouette	Mean (year)	Label (LSI)
#0 short-chain fatty acids	0.626	2017	Gut microbiota; isolated systolic hypertension; isolated diastolic hypertension; human urinary microbiome; urinary system-related diseases; short-chain fatty acids; olfactory receptor; microbial metabolites; maternal high fructose diet; hypothalamic paraventricular nucleus
#1 ulcerative colitis	0.564	2019	Gut microbiota; oxidative stress; antioxidant peptides; plant-based food peptides; non-alcoholic fatty liver disease; gut microbiome; fecal microbiota transplantation; microbial detoxification; food arsenic; arterial stiffness
#2 bioactive compounds	0.612	2017	Gut microbiota; short chain; gut model; *Lactiplantibacillus plantarum*; hypertensive rats; cardiovascular disease; fermented dairy products; systems biology; dietary patterns; obesity treatment
#3 metabolic syndrome	0.686	2016	Gut microbiota; metabolic syndrome; developmental origins; fatty acid; short chain; insulin resistance; glucose metabolism; non-absorbable antibiotics; arterial stiffness; animal models
#4 portal hypertension	0.811	2008	Gut microbiota; obstructive sleep apnea; apolipoprotein e-deficient; chronic intermittent hypoxia; systems biology; portal hypertension; intestinal permeability; molecular mimicry; primary biliary cirrhosis; liver damage
#5 trimethylamine n-oxide	0.64	2018	Gut microbiota; chronic kidney disease; blood pressure; fibroblast growth factor; metabolic acidosis; cardiovascular diseases; gut bacteria; hydrogen sulfide; fatty liver disease; liver transplantation
#6 cardiovascular disease	0.815	2011	Cardiovascular disease; cardiometabolic health; precision nutrition; caloric restriction; dietary intervention; gut microbiota; short-chain fatty acids; histone acetylation; protein-coupled receptors; diet therapy
#7 gut microbiota	0.816	2011	Portal hypertension; bacterial translocation; intestinal microflora; mesenteric lymph nodes; hepatic immunological tolerance; gut microbiota; molecular docking; network pharmacology; insulin resistance; imidazole propionate
#8 lactic acid bacteria	0.942	2005	Lactic acid bacteria; fermented foods; aminobutyric acid; *weissella confusa* g2; metabolic syndrome; gut microbiota; converting enzyme; oxidative stress; yeast protein hydrolysate; gut microbiome
#9 blood pressure	0.904	2010	Gut microbiota; obstructive sleep apnea; trimethylamine n-oxide; renal denervation; arterial stiffness; blood pressure; olfactory receptors; arterial stiffness; immune homeostasis; endothelial dysfunction

In summary, keyword clustering provides a detailed deconstruction of the research landscape on hypertension and gut microbiota, spanning fundamental mechanisms (e.g., #7 gut microbiota), metabolite functions (e.g., #5 trimethylamine N-oxide), clinical disease associations (e.g., #6 cardiovascular disease), and intervention strategies (e.g., #2 bioactive compounds). The high silhouette values (many >0.7) attest to the reliability of these clusters and support the identification of research gaps—such as limited attention to certain populations or novel interventions—thus enabling precise prediction of future trends and facilitating the transition from fragmented studies toward a more systematic and targeted research paradigm.

### 3.4 Bibliometric analysis of keyword citation bursts

**Figure 10:**
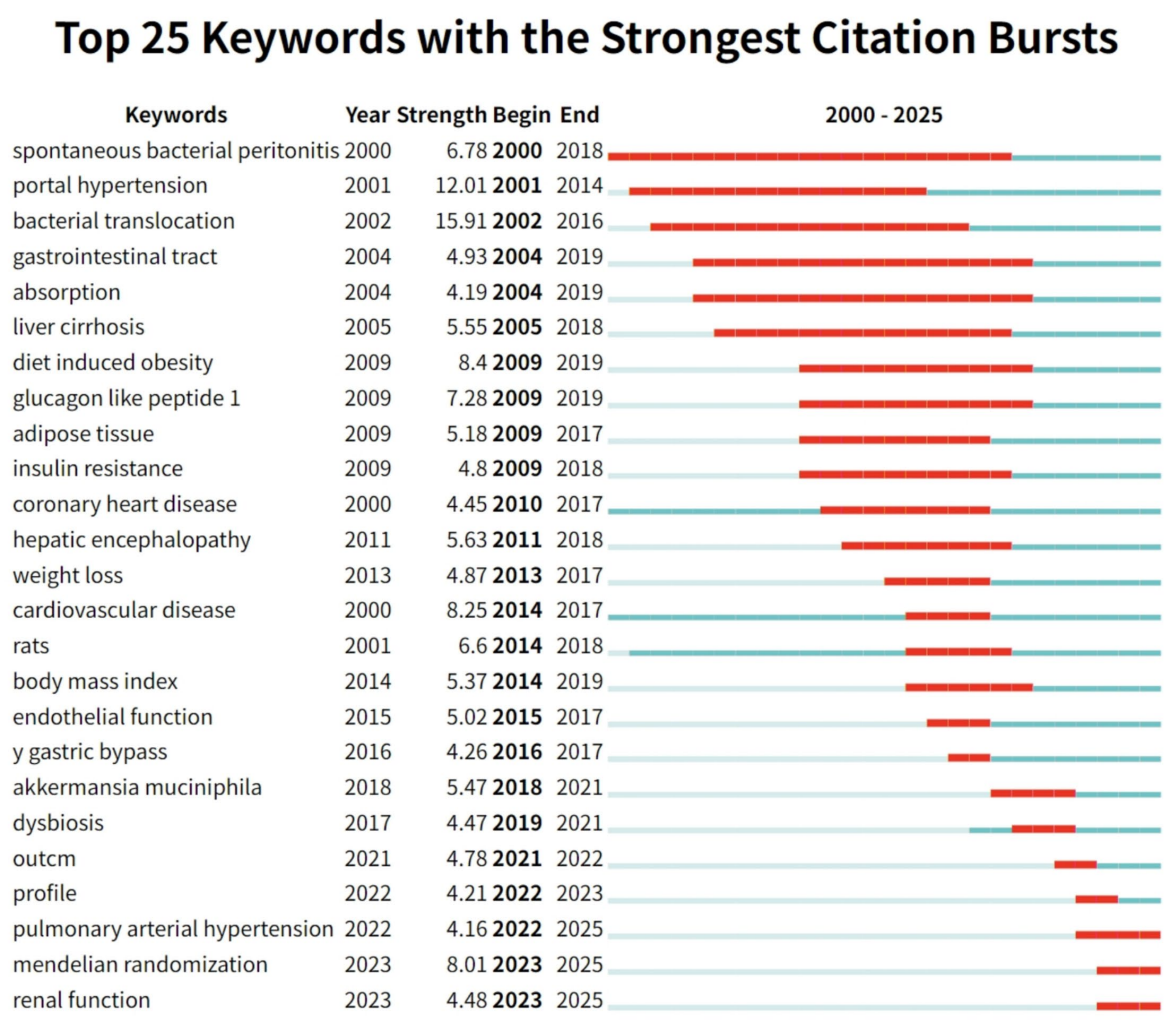
The top 25 keywords with the strongest citation bursts are displayed. Notably, since 2022, three keywords—“pulmonary arterial hypertension,” “Mendelian randomization,” and “renal function”—have emerged as new citation bursts.

Keyword citation bursts identified as periods when specific keywords experience a rapid increase in citation frequency provide valuable insights into emerging research hotspots and developmental trends within a field (Chen, 2006). Using the Top 25 keyword citation burst map generated by CiteSpace (Figure 10) we systematically traced the evolving research frontiers shifting hotspots and future directions in studies on hypertension and gut microbiota. As illustrated in Figure 10 the top 25 keywords with the strongest burst intensities from 2000 to 2025 can be grouped into three chronological stages:

Early-stage citation bursts (2000–2010) reflect persistent attention to foundational mechanisms. Keywords such as “spontaneous bacterial peritonitis” (burst intensity 6.78, 2000–2018) and “portal hypertension” (12.01, 2001–2014) were prominent, highlighting the field’s initial focus on the classical pathological pathway of gut microbiota translocation leading to infection, liver disease, and subsequent blood pressure abnormalities. Related terms like “bacterial translocation” (15.91, 2002–2016) and “liver cirrhosis” (5.55, 2005–2018) further established the basis for studies on gut–liver–circulatory system interactions, underscoring the long-lasting influence of classic mechanistic research. In the mid-stage (2009–2015), research focus shifted toward metabolic regulation and intervention strategies. In the metabolic domain, keywords such as “diet induced obesity” (8.4, 2009–2019), “glucagon like peptide 1” (7.28, 2009–2019), and “insulin resistance” (4.8, 2009–2018) experienced notable bursts, reflecting a surge in research linking gut microbiota, metabolic disorders, and hypertension amidst the rise of obesity and diabetes globally. Meanwhile, “bariatric bypass” (4.26, 2016–2017) appeared as a short-lived burst, signaling emerging interest in surgical interventions that affect gut microbiota and blood pressure, and indicating the nascent trend toward microbiota-targeted interventions. In the recent stage (2017–2025), keyword bursts highlight significant innovation in precise mechanistic research and personalized interventions. Bursts such as “*Akkermansia muciniphila*” (5.47, 2018–2021) and “dysbiosis” (4.47, 2019–2021) demonstrate a shift from general descriptions of the microbiota to detailed investigations of specific functional genera (e.g., Akkermansia) in blood pressure regulation. Methodological innovations are evidenced by the appearance of “Mendelian randomization” (8.01, 2023–2025), marking an evolution from observational studies to robust causal inference in microbiota–hypertension research. Clinically, the burst of “pulmonary arterial hypertension” (4.16, 2022–2025) signals expanding interest in the role of gut microbiota in rare forms of hypertension and the progression toward more precise disease subtyping and personalized prevention and treatment strategies.

In summary, analysis of keyword citation bursts reveals the evolutionary trajectory of research on hypertension and gut microbiota: from classical gut–liver axis mechanisms, through metabolic and intervention-focused studies, toward precise microbiota modulation and clinical subtyping. Emerging burst keywords such as “Mendelian randomization” and “Akkermansia” inject fresh momentum into the field, highlight unresolved issues such as population heterogeneity and drug–microbiota interactions, and help chart the path toward future breakthroughs and the development of mechanism-based, precision clinical translation.

### 3.5 Analysis of keyword timeline and peaks visualization

The keyword timeline visualization maps the evolution, interconnectedness, and research trends of topics related to hypertension and gut microbiota over time. By aligning themes along a chronological axis and clustering them by topic, this approach provides valuable insights into how knowledge has disseminated and research directions have shifted in the field (Chen, 2006).

**Figure 11:**
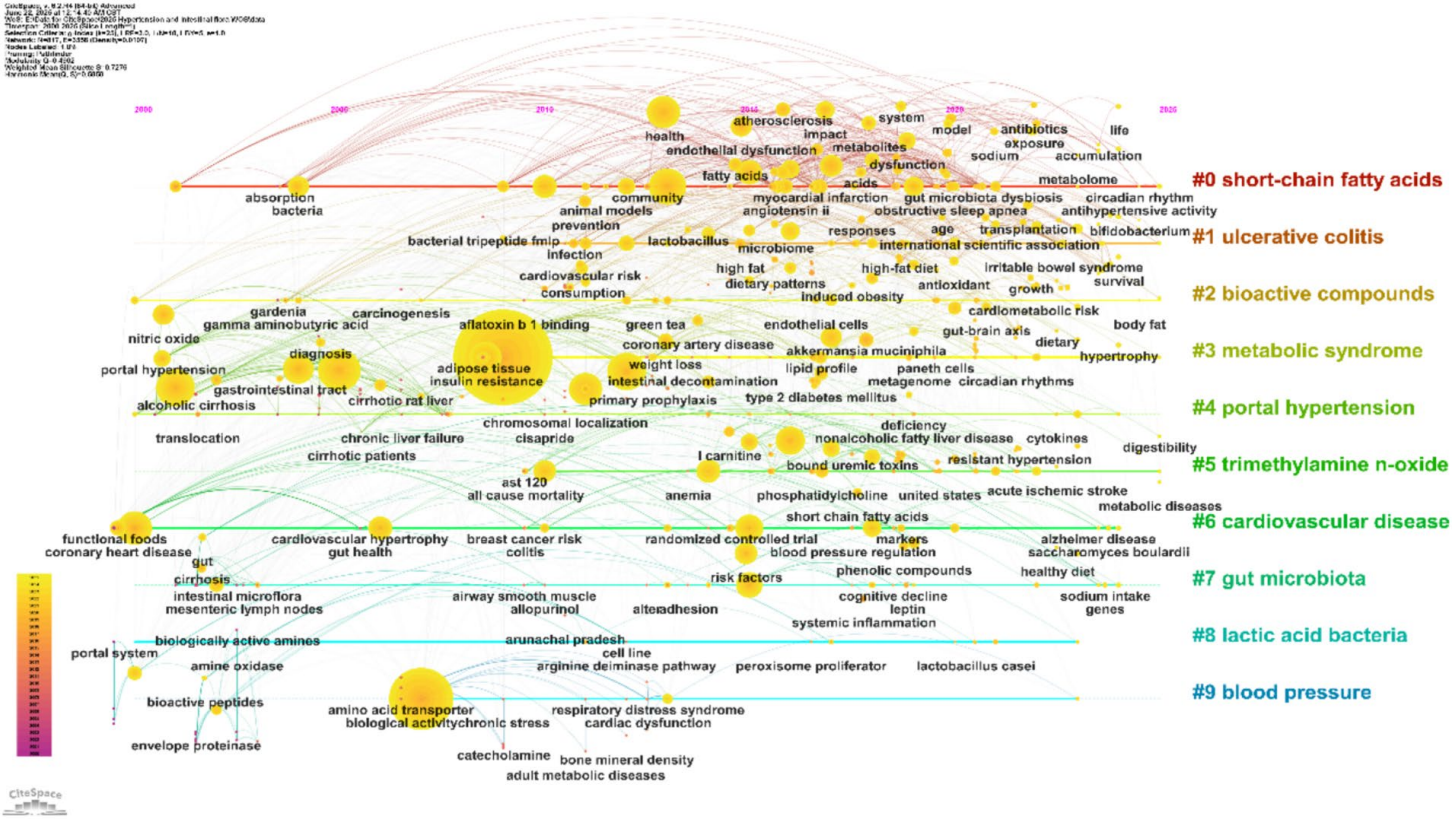
Keyword timeline. The horizontal axis represents time, with different colored regions indicating research clusters. Node size corresponds to keyword frequency, and links between nodes illustrate thematic relationships.

As shown in Figure 11, the timeline arranges keywords across the horizontal axis (years 2000–2025) and groups them vertically by research clusters (#0 to #9). Each node’s size indicates the frequency of keyword co-occurrence, reflecting levels of research activity, while the strength of connections between nodes represents the degree of interrelation among topics. Cluster labels, extracted using the Latent Semantic Indexing (LSI) algorithm, summarize the core research foci for each stage.

During the foundational period from 2000 to 2010, clusters centered on #4 “portal hypertension” and #8 “lactic acid bacteria” emerged as key nodes. Frequent associations with keywords such as “portal hypertension,” “bacterial translocation,” and “liver cirrhosis” reflect an early focus on the classical gut–liver axis and its role in liver injury and blood pressure dysregulation. Concurrently, “lactic acid bacteria” and “fermented foods” were prominent, highlighting early exploration of probiotic interventions and establishing a basis for subsequent studies on metabolic regulation. From 2011 to 2020, research expanded toward both metabolic regulation and clinical manifestations. Strong networks formed around “metabolic syndrome,” “insulin resistance,” and “trimethylamine n-oxide (TMAO),” corresponding to a surge in studies on the links between gut microbiota, metabolic disturbances, and hypertension in response to the global rise of metabolic diseases. TMAO, as a key microbial metabolite, emerged as a central connector between metabolic abnormalities and blood pressure regulation. Additionally, keywords like “ulcerative colitis,” “cardiovascular disease,” “oxidative stress,” and “fecal microbiota transplantation” became increasingly interrelated, indicating deeper investigation into the interplay between inflammatory bowel diseases, cardiovascular complications, and gut microbiota—thus driving the field toward translational clinical research. Since 2021, research has increasingly focused on precise modulation of the microbiota and integration of multi-omics approaches. Core associations have formed among “short-chain fatty acids (SCFAs),” “gut microbiota,” “endothelial dysfunction,” and “blood pressure regulation,” revealing molecular pathways such as the gut–vascular axis in blood pressure control. The field has moved from general descriptions of the microbiota to studies targeting specific metabolites and mechanisms. Frequent co-occurrence of keywords such as “bioactive compounds,” “dietary patterns,” and “hypertensive rats,” alongside the emergence of “precision nutrition,” highlights an ongoing shift toward personalized microbial interventions and tailored dietary–microbiota–blood pressure strategies.

Overall, the keyword timeline provides a systematic overview of the research evolution in hypertension and gut microbiota, tracing a path from initial gut–liver axis studies, through expansion into metabolic and clinical domains, to current precision and personalized intervention approaches. The visualization also highlights gaps in cross-population and cross-technology research, underscoring future opportunities for methodological innovation and clinical translation. This comprehensive mapping supports the transition from foundational explorations to integrated clinical applications.

The trend plot visualizes research activity by mapping time on the horizontal axis and research clusters on the vertical axis. The shape of each curve intuitively reflects changes in research intensity and developmental dynamics across clusters within the field of hypertension and gut microbiota. This graphical approach provides a valuable basis for understanding the temporal pace of research and identifying emerging hotspots (Chen, 2017).

**Figure 12:**
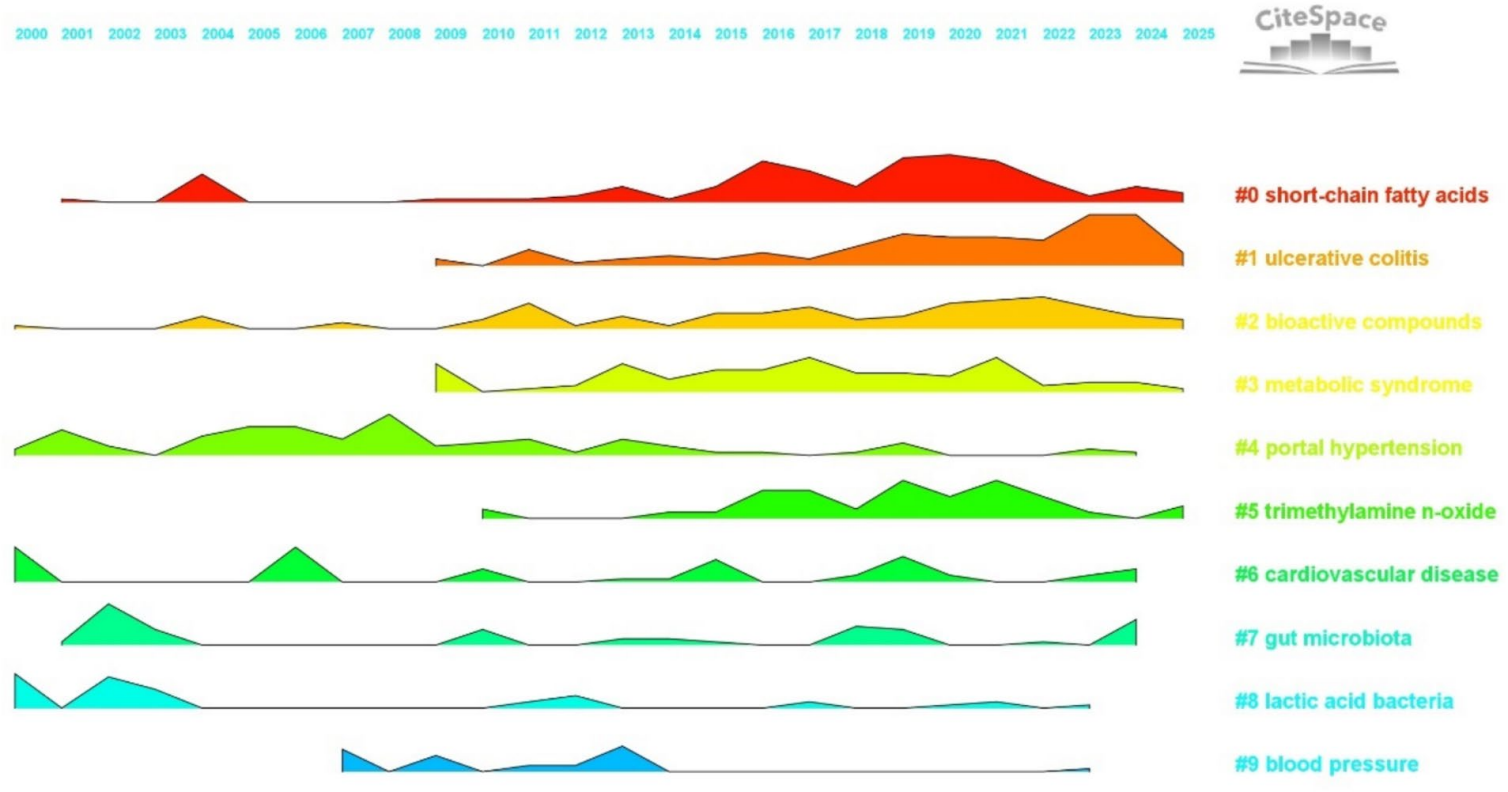
Landscape view. The horizontal axis represents the years 2000 to 2025, with different colored regions denoting distinct research clusters. The height of each peak indicates the intensity of research activity for each cluster in a given year.

As illustrated in Figure 12, each colored curve represents a specific keyword cluster (#0 to #9). The height of each peak indicates the intensity of research activity (measured by keyword co-occurrence), while the width represents the duration of sustained interest. Fluctuations in curve shape reveal trends and transitions in research focus. By analyzing these patterns, it is possible to trace the lifecycle of each topic, including phases of emergence, growth, maturity, and decline, thereby capturing the dynamic evolution of the field.

Analysis of the temporal trends in cluster activity reveals distinct patterns. Foundational clusters show long-term stability, serving as the basis of the field. For example, cluster #4, “portal hypertension” (green curve), displayed a prominent peak between 2000 and 2010, with continued, albeit fluctuating, activity thereafter. This reflects the enduring influence of classical gut–liver axis mechanisms, with early research focusing on connections between “bacterial translocation,” “liver cirrhosis,” and blood pressure regulation. Similarly, cluster #8, “lactic acid bacteria” (light blue curve), maintained steady activity from 2000 to 2015, centering on “fermented foods” and “probiotics” to explore microbial intervention strategies—providing a framework for subsequent studies in targeted microbiota modulation. In the mid-developmental stage, certain clusters experienced marked increases in research intensity, driving significant advances in the field. Cluster #3, “metabolic syndrome” (yellow curve), showed a sustained rise in activity from 2009 to 2020, peaking between 2015 and 2020, paralleling a global surge in research linking gut microbiota, metabolic disorders, and hypertension. Frequent co-occurrence of keywords like “insulin resistance” and “diet induced obesity” underscores the central role of metabolic mechanisms in microbiota-mediated blood pressure regulation. Likewise, cluster #5, “trimethylamine N-oxide (TMAO)” (light green curve), steadily gained prominence after 2010, with a notable peak from 2015 to 2022. Research in this cluster emphasized the connection between TMAO, cardiovascular disease, and hypertension, establishing TMAO as a critical link between microbial metabolites and clinical outcomes, and advancing the field from basic associations to mechanistic insights. Recently, emerging and innovative clusters have rapidly gained momentum, shaping the research frontier. Cluster #1, “ulcerative colitis” (orange curve), exhibited a sharp rise in activity after 2010, peaking between 2020 and 2025. This trend highlights the growing focus on the gut microbiota mechanisms underlying inflammatory bowel diseases and hypertension, with frequent linkage to keywords such as “oxidative stress” and “fecal microbiota transplantation.” This represents a breakthrough in understanding gut–immune–cardiovascular system interactions and fosters further clinical translation. Cluster #0, “short-chain fatty acids” (red curve), has sustained high activity from 2015 to 2025, with research clustering around “SCFAs,” “endothelial function,” and “blood pressure regulation.” These studies have elucidated molecular pathways by which SCFAs influence blood pressure through the gut–vascular axis, marking a transition from broad microbiota research to targeted exploration of key metabolites and mechanisms.

In summary, the trend plot offers a systematic visualization of research evolution in hypertension and gut microbiota, highlighting the foundational role of classical clusters, the mechanistic expansion during the mid-developmental stage, and the innovative breakthroughs driven by emerging clusters. Future research should focus on precision modulation and clinical translation, fostering greater collaboration across clusters and integration of advanced technologies. Such efforts will transform the field from fragmented studies to systematized innovation, supporting the establishment of a closed-loop pathway from mechanistic discovery to clinical intervention.

## 4 Discussion

### 4.1 General information

Using CiteSpace and VOSviewer, this study systematically analyzes the research landscape of hypertension and gut microbiota, highlighting major trends and emerging frontiers. Data were obtained from the Web of Science Core Collection, and after Boolean retrieval and deduplication, a total of 2,827 articles published between 2000 and 2025 were included. The publication trend in this field shows three distinct phases: a period of slow accumulation from 2000 to 2013 (less than 50 articles per year), a phase of rapid growth from 2014 to 2019 (up to 200 articles per year), and a plateau of high output from 2020 to 2025 (250–450 articles annually). This rising trend is primarily driven by advances in multi-omics technologies and increased demand for clinical interventions.

China (918 publications) and the United States (676 publications) are the leading contributors, together accounting for 56% of global output. Highly productive institutions include Zhejiang University in China and the University of Florida in the United States. Prominent research groups comprise the team led by Yang Tao at the University of Florida (focused on metabolic mechanisms), the group led by Raizada Mohan K. (emphasizing clinical translation), and Tain You-Lin’s team at Chang Gung University in Taiwan (specializing in probiotic interventions). International collaboration in this field is characterized by a network pattern led by China and the United States, with broad participation from multiple countries. The United States, Germany, and Spain focus on microbiota metabolic mechanisms, while China, Japan, and South Korea emphasize population-based cohorts and clinical translation.

Key research topics revolve around gut microbiota and blood pressure, forming a framework that links metabolic regulation, complication associations, and intervention strategies. Metabolites such as short-chain fatty acids and trimethylamine N-oxide are critical nodes of interest. Since 2017, Mendelian randomization and *Akkermansia muciniphila* have emerged as prominent research hotspots, propelling the field toward precise mechanistic understanding and personalized interventions. Looking ahead, future research should enhance interdisciplinary collaboration, focus on ethnicity-specific microbial characteristics and microbe–drug interactions, and accelerate the clinical translation of microbiota-targeted therapies.

### 4.2 Dynamic evolution of the research field

The development of research on hypertension and gut microbiota has undergone three distinct phases. The first, from 2000 to 2013, was characterized as a foundational exploratory period. During this phase, topics such as “portal hypertension” (citation burst strength: 12.01, 2001–2014) and “bacterial translocation” (15.91, 2002–2016) emerged as focal points, reflecting early interest in the classic gut–liver axis pathophysiology. A pivotal milestone was reached in 2013 when Pluznick et al. demonstrated in Nature that gut microbiota-derived metabolites can regulate host blood pressure, establishing the theoretical basis for the “microbiota–metabolism–blood pressure” link. Research in this era was largely descriptive, with annual publications rarely exceeding 50. Most studies focused on associations between bacterial translocation, liver injury, and inflammatory responses. For instance, it was shown that dysbiosis in patients with liver cirrhosis could induce abnormal blood pressure via endotoxemia (Efremova et al., 2024). Methodologically, work was constrained by traditional microbial culture techniques, limiting investigations to microbial diversity rather than metabolic mechanisms. The second stage, from 2014 to 2019, marked a period of mechanistic expansion. Notable research bursts centered on “diet-induced obesity” (8.4, until 2019) and “insulin resistance” (4.8, until 2018), signifying a shift toward exploring the interplay between gut microbiota, metabolic disorders, and hypertension. Advances in multi-omics technologies fueled a sharp rise in publication volume, from roughly 30 to nearly 200 articles per year. Key discoveries included the role of short-chain fatty acids (e.g., butyrate) in modulating vascular endothelial function via GPR43 receptors, and trimethylamine N-oxide (TMAO) promoting atherosclerosis through the FMO3 pathway (Kimura et al., 2013; Yu et al., 2021; Kong et al., 2024). Following 2017, “*Akkermansia muciniphila*” (5.47, until 2021) emerged as a new hotspot, with studies showing its capacity to repair the gut barrier and reduce endotoxemia, thereby improving blood pressure. This period also saw a deepening focus from overall gut microbiota profiles to functional bacterial taxa. More than 70% of studies utilized animal models, and early clinical investigations began to assess the efficacy of probiotics such as *Lactobacillus plantarum* in pre-hypertensive populations (Khalesi et al., 2014). The most recent phase, from 2020 to 2025, is characterized by precision and deepening mechanistic insights. The emergence of “Mendelian randomization” (8.01, since 2023, projected through 2025) has addressed the limitations of traditional observational studies by establishing a causal link between reduced gut microbiota diversity and increased hypertension risk (He et al., 2023). “Pulmonary arterial hypertension” (4.16, through 2025) has also become a new research hotspot, implicating the gut–lung axis in the pathogenesis of specific hypertension subtypes (Prisco et al., 2025). The research paradigm has shifted toward precise mechanistic elucidation and personalized interventions, including molecular modeling of blood pressure regulation based on microbiota metabolites (short-chain fatty acids, bile acids) and the use of AI algorithms to predict individual responses to microbiota-targeted therapies (Verhaar et al., 2020). Annual publications have stabilized at 250–450, with clinical research increasingly focusing on microbiota transplantation and drug–microbiota interactions, such as the effects of RAAS inhibitors on gut microbial composition (Dong et al., 2022). This period is marked by pronounced interdisciplinary integration, particularly among microbiome science, cardiovascular medicine, and nutrition, promoting the elucidation of the tripartite regulatory network among microbiota, pharmaceuticals, and the host.

### 4.3 International collaboration and research power structure

In the field of hypertension and gut microbiota research, China (918 publications) and the United States (676 publications) serve as dual engines, together accounting for 56.4% of global output. This has established a landscape where China and the US lead, with participation from multiple other countries. China, leveraging large patient cohorts and policy support, excels in population-based and translational clinical studies, particularly focusing on probiotic interventions. The US, with its strong foundation in microbiological research, leads in elucidating microbial metabolic mechanisms, driving breakthroughs in fundamental theories such as the short-chain fatty acids–gut–vascular axis. Although Germany and Spain publish fewer articles, they boast a higher proportion of highly cited papers (12.3 and 11.7% respectively), highlighting their research quality and focus on mechanisms like oxidative stress. Emerging economies, such as India and Brazil, are gradually integrating into the research network, though resource distribution remains uneven.

International collaboration follows a “core–periphery” model. The US, Germany, and Spain have formed a cluster focused on microbial metabolic mechanisms, while China, Japan, and South Korea have established an East Asian alliance for clinical translation. China and the US also lead several multicenter studies; for example, the University of Florida and Zhejiang University have jointly investigated the role of *Akkermansia muciniphila* in blood pressure regulation. However, cross-regional collaboration remains limited, with only 15% of teams spanning multiple regions, indicating a persistent geographic concentration. Institutional research displays clear regional clustering. In China, universities have formed a collaborative network in the Yangtze River Delta, focusing on population-specific microbiota mechanisms. Institutions in Taiwan specialize in probiotic interventions supported by dedicated funding. American universities have developed a southeastern collaboration network with an emphasis on metabolomics and causal inference. Newer institutions in Brazil and India are joining the network through international collaborations, although their ties with core institutions are still relatively weak.

Among leading researchers, the team led by Yang Tao at the University of Florida has constructed a model for gut microbiota metabolite regulation of blood pressure, which has been cited 664 times—the highest in the field (Yang et al., 2015). The group led by Tain You-Lin at Chang Gung University in Taiwan demonstrated that *Lactobacillus plantarum* can lower blood pressure by modulating the gut barrier, advancing clinical translation (Chen et al., 2022). Li Jing’s team at Capital Medical University has analyzed the relationship between gut microbiota and arterial stiffness using metagenomic sequencing (Li J. et al., 2023). Going forward, it is essential to strengthen international collaboration and interdisciplinary integration, to overcome barriers in mechanistic translation and to develop universally applicable gut microbiota-based intervention strategies for hypertension prevention and control.

### 4.4 Research hotspots and mechanistic innovations

#### 4.4.1 Metabolite-mediated mechanisms of blood pressure regulation

Short-chain fatty acids (SCFAs), such as acetate, propionate, and butyrate, are produced by the fermentation of dietary fiber by gut microbiota. They have attracted significant attention due to their bidirectional effects on blood pressure regulation. SCFAs can lower blood pressure by activating G protein–coupled receptors (GPCRs), specifically GPR41 and GPR43 found in the gut and vascular walls. Activation of these receptors suppresses renin release and reduces sympathetic nervous system activity. Pluznick et al. (2013, Nature) demonstrated that deleting the GPR41 gene in mice eliminates blood pressure sensitivity to SCFAs, resulting in a 15–20 mmHg increase in systolic pressure (Kimura et al., 2011; Kaye et al., 2020). Additionally, butyrate can inhibit histone deacetylase (HDAC) activity and enhance endothelial nitric oxide synthase (eNOS) expression, which leads to increased nitric oxide (NO) release and vasodilation. Bridgeman et al. found that butyrate intervention elevated vascular NO production by 40% and reduced mean arterial pressure by 18% in spontaneously hypertensive rats (SHR) (Bridgeman et al., 2020). However, clinical findings remain inconsistent. In the HELIUS cohort study (*n* = 4,672), Verhaar et al. (2020, European Heart Journal) observed that each standard deviation increase in fecal acetate was associated with a 3.1 mmHg rise in systolic blood pressure (*p* < 0.01). This may reflect differences in SCFA absorption, as hypertensive patients often display higher expression of intestinal SCFA transporters (e.g., SMCT1), leading to elevated plasma SCFA levels but lower fecal excretion. Notably, the abundance of SCFA-producing bacteria, such as Roseburia, is negatively correlated with blood pressure (Verhaar et al., 2020). Yang et al. (Hypertension) reported a significant decrease in gut microbiota diversity and an altered Firmicutes/Bacteroidetes (F/B) ratio in hypertensive patients (Yang et al., 2015). These findings suggest that the regulatory effects of SCFAs on blood pressure are governed by a complex interplay among bacterial composition, host absorption efficiency, and metabolic pathways. Kasahara et al. (Nature Microbiology) showed that mice colonized with Roseburia and fed a high-fiber diet exhibited reduced atherosclerosis and lower inflammatory markers, but this protective effect was lost with a low-fiber diet. Mechanistically, Roseburia ferments dietary fiber to produce butyrate, which activates GPR41, suppresses renin release, and reduces sympathetic nervous system activity (Kasahara et al., 2018).

Trimethylamine N-oxide (TMAO), a metabolite produced by gut microbiota from choline and carnitine, contributes to elevated blood pressure mainly by activating the NLRP3 inflammasome and promoting atherosclerosis. Li et al. (2017, European Heart Journal) found that TMAO activates the NLRP3 inflammasome via the ROS/NF-κB pathway, increasing IL-1β secretion and causing endothelial dysfunction. In rats, a four-week TMAO intervention (120 μM/d) reduced aortic endothelium-dependent relaxation by 35% and raised systolic blood pressure by 25 mmHg (Li et al., 2017). Clinically, Tang et al. reported that subjects with the highest plasma TMAO (>6.18 μM) had a 1.9-fold higher risk of hypertension compared to those with the lowest levels (<2.72 μM; HR = 2.91, *p* < 0.001) (Koeth et al., 2013). From a microbiota–host perspective, Wang et al. (Frontiers in Cardiovascular Medicine) used Mendelian randomization (*n* = 2076) to show that TMAO and its precursor carnitine were positively associated with systolic blood pressure, while choline and betaine were not significantly related (Wang et al., 2022).

Bile acids (BAs) and trimethylamine (TMA) also work together in regulating blood pressure and maintaining gut barrier integrity. Primary bile acids are converted by gut microbiota into secondary bile acids such as deoxycholic acid, which can activate the FXR receptor to modulate lipid metabolism and indirectly affect cardiovascular risk (Wang Q. et al., 2023; Ren et al., 2024). Pathak et al. demonstrated that administering an FXR agonist to SHR rats for 8 weeks lowered systolic blood pressure by 22 mmHg (Singh et al., 2018). Jaworska et al. observed that TMA increased colonic permeability in SHR rats, a phenomenon likely related to hemodynamic changes or inflammation (Jaworska et al., 2017).

Bibliometric analysis further underscores the centrality of these mechanisms. Keyword clustering (Figure 9) identified “short-chain fatty acids” and “trimethylamine N-oxide” as core research clusters, each with a silhouette value greater than 0.64, reflecting a high degree of focus in mechanistic studies. Burst analysis (Figure 10) showed that “trimethylamine N-oxide” had a burst strength of 5.47 from 2018 to 2025, highlighting TMAO as a persistent research frontier and emphasizing its prominence in the field.

Overall, metabolite-mediated regulation of blood pressure is bidirectional: while SCFAs generally lower blood pressure through the GPCR and HDAC pathways, host absorption efficiency may modify these effects. In contrast, TMAO consistently increases blood pressure through inflammatory activation. Future research should integrate host metabolic phenotypes with microbiota functional genomics to develop more precise intervention strategies.

#### 4.4.2 Gut microbiota–host interactions and disease associations

In obstructive sleep apnea (OSA), intermittent hypoxia compromises gut barrier integrity, allowing bacterial lipopolysaccharide (LPS) to enter systemic circulation and trigger inflammation. This gut–heart axis is a crucial mechanism linking OSA to hypertension. Durgan et al. demonstrated this by transplanting fecal microbiota from OSA patients into germ-free mice. Recipient mice exhibited a 40% decrease in intestinal mucosal ZO-1 protein expression, a 2.5-fold increase in serum LPS, and a 25 mmHg rise in systolic blood pressure. These effects were reversed by the TLR4 inhibitor TAK-242 (Durgan et al., 2016). *Akkermansia muciniphila* abundance is positively correlated with gut barrier integrity, and Mendelian randomization analysis indicates that each standard deviation increase in the abundance of Verrucomicrobia (the phylum to which *A. muciniphila* belongs) is associated with a 17% reduction in hypertension risk (He et al., 2023). Mechanistically, LPS binds to TLR4, activating the NF-κB pathway and promoting the secretion of IL-6 and TNF-α, which contribute to endothelial dysfunction. Wilck et al. showed that a high-salt diet tripled colonic TLR4 expression in mice, and TLR4 antagonists restored normal blood pressure.

SCFAs play a critical role in modulating endothelial function within the gut–vascular axis, with the butyrate-NO pathway being of particular interest. Cluster #0 (silhouette value 0.626) highlights the mechanisms by which SCFAs regulate blood pressure via this axis. Pluznick et al. showed that butyrate activates the intestinal epithelial receptor Olfr78, suppressing renin release while simultaneously promoting NO-mediated vasodilation by upregulating eNOS expression through HDAC inhibition [3]. Clinical studies support this mechanism, demonstrating a strong correlation between reduced plasma butyrate levels and impaired endothelial function (e.g., impaired flow-mediated dilation [FMD]) in hypertensive patients. Butyrate supplementation can improve gut microbiota composition and vascular function, thereby lowering blood pressure (Sun et al., 2022; Tilves et al., 2022).

In cardiovascular disease (cluster #6, silhouette value 0.815), dysbiosis influences myocardial collagen deposition through SCFA receptor (GPR43)-mediated histone acetylation. Mendelian randomization studies by Wang et al. suggest that microbial metabolites indirectly reduce cardiovascular risk by suppressing inflammation and improving endothelial function (Wang Q. et al., 2023). In chronic kidney disease (cluster #5, silhouette value 0.64), a gut–kidney–blood pressure vicious cycle exists. Impaired renal function leads to the accumulation of uremic toxins, exacerbating dysbiosis. TMAO accumulation further damages renal tubules, and elevated TMAO levels are negatively correlated with estimated glomerular filtration rate (eGFR) in CKD patients (Wang et al., 2024, 2025). Tang et al. found that elevated TMAO levels in CKD patients are associated with the progression of renal insufficiency (Tang et al., 2015; Pelletier et al., 2019). In childhood hypertension, dysbiosis may influence blood pressure through inflammation and altered SCFA metabolism, with enrichment of specific genera (e.g., Bacteroides) correlating with increased systolic blood pressure (Al Khodor et al., 2017; Differding et al., 2020).

Keyword cluster analysis (Figure 9) revealed an association between cluster #1 (ulcerative colitis) and “oxidative stress” and “arterial stiffness” (correlation coefficient = 0.42) confirming the role of inflammatory pathways in vascular damage. Cluster #6 (cardiovascular disease) encompassed “precision nutrition” and “histone acetylation,” reflecting complex interactions between metabolism and epigenetics. Burst analysis (Figure 10) identified “pulmonary arterial hypertension” as an emerging research area within the hypertension field with a high burst strength of 4.16 from 2022 to 2025.

The gut microbiota–host axis bidirectionally regulates blood pressure through the “gut barrier–LPS–TLR4” inflammatory pathway and the “SCFAs–endothelial function” metabolic pathway. Studies on associated complications reveal that reduced microbial diversity drives myocardial fibrosis in cardiovascular disease; TMAO accumulation creates a gut–kidney vicious cycle in chronic kidney disease; and age-specific microbial markers, such as decreased Blautia, are present in childhood hypertension. Future research should focus on developing targeted microbiota intervention strategies for different complications.

#### 4.4.3 Targeted intervention strategies

Probiotics and prebiotics show significant clinical promise for managing hypertension. Strain-specific effects have been observed; for instance, *Lactobacillus plantarum* regulates blood pressure through the gut–brain axis by several mechanisms. It strengthens the gut barrier by upregulating proteins such as ZO-1 and Occludin and reducing serum I-FABP, thereby decreasing intestinal permeability and endotoxemia. It also promotes the growth of butyrate-producing bacteria like Blautia and Roseburia, enhancing vasodilation via GPR43 activation and suppressing the renin–angiotensin system. Additionally, it reduces pro-inflammatory cytokines (IL-6, TNF-α) and increases anti-inflammatory factors (IL-10), ultimately improving endothelial health and lowering blood pressure (Duarte Luiz et al., 2025). *Lactobacillus rhamnosus* GG (LGG) has also demonstrated antihypertensive effects in high-salt diet rat models, restoring systolic pressure by increasing villus-to-crypt ratio and lowering serum LPS (Zaharuddin et al., 2025). Among prebiotics, inulin improves vascular function by modulating the gut microbiota (e.g., increasing Bifidobacterium abundance) and SCFA production, showing blood pressure–lowering effects in animal models (Azuma et al., 2023; Shi et al., 2023; Mahgoup, 2025). Jama et al. recommend that hypertensive women consume more than 28 g/day and men more than 38 g/day of dietary fiber; every 5 g increase in daily fiber intake is estimated to reduce systolic pressure by 2.8 mmHg and diastolic by 2.1 mmHg (Jama et al., 2024). In clinical practice, personalizing probiotic and prebiotic selection and dosage according to gut microbiota composition and metabolic status may enhance antihypertensive efficacy. Furthermore, combined use of probiotics and prebiotics may become a vital future strategy in hypertension management.

Microbiota transplantation and dietary interventions also play important roles in blood pressure regulation. In disease models of OSA-related hypertension, fecal microbiota transplantation (FMT) from OSA patients (with or without hypertension) into C57BL6J mice resulted in significant increases in systolic blood pressure and aortic injury. This is likely due to OSA-associated dysbiosis impairing gut barrier function (evidenced by decreased ZO-1 and Occludin), which activates the LPS/TLR4/NF-κB inflammatory pathway and vascular injury (Zhang et al., 2025). Supplementation with inactivated *Akkermansia muciniphila* (10^10^ CFU/day) has been shown to increase insulin sensitivity by 28.6% (*p* = 0.002) in overweight or obese individuals with insulin resistance, likely by repairing the gut barrier and enhancing GLP-1 secretion (Depommier et al., 2019). Regarding dietary patterns, the ATTICA prospective cohort study followed 3,042 adults without hypertension for 20 years and found that those with the highest adherence to a Mediterranean diet had a hypertension incidence of only 8.7%, compared to 35.5% in those with the lowest adherence. Each one-point increase in MedDietScore corresponded to a 7% reduction in hypertension risk (Georgoulis et al., 2024).

Integrated, multi-target interventions have also been explored. Ning et al. reported that hypertensive patients on high-calorie diets exhibited reduced abundance of Lachnospiraceae, which correlated with decreased serum HDL-C (Lan et al., 2022). Li et al. demonstrated that butyrate supplementation lowers blood pressure in rats by modifying gut microbiota and activating brown adipose tissue. In adults, this effect is related to SCFA receptor activity, but pediatric data are lacking (Li et al., 2024). A 2018 study found that BMI and body fat percentage have limited predictive value for childhood hypertension (AUC: 0.64–0.70) (Renying et al., 2018). These findings support the relevance of gut–metabolic regulation in hypertension and highlight the need to clarify childhood-specific mechanisms.

Supporting data show that keyword cluster analysis (Figure 9) identifies “bioactive compounds” (cluster #2, silhouette value 0.612) including “fermented dairy products” and “dietary patterns,” underscoring the central role of dietary interventions in cardiovascular health. Additionally, burst analysis (Figure 10) reveals that “Mendelian randomization” had a burst strength of 8.01 from 2023 to 2025, providing new methods for causal inference in microbiota-targeted interventions.

Targeted microbiota interventions for hypertension have developed along three main lines: (1) precision use of probiotics and prebiotics to modulate gut microbiota for blood pressure control; (2) targeting key metabolic pathways, such as those involving short-chain fatty acids and trimethylamine N-oxide; and (3) integrated, multi-target approaches combining dietary, pharmacological, and microbiota-based strategies. Future research must address two key challenges: individual response variability—affected by genetic background, age, and baseline microbiota composition (with clinical data suggesting stronger prebiotic responses in Asian populations compared to European populations)—and long-term safety, as the ecological impacts of probiotic colonization and the risks of microbiota transplantation require validation in large-scale randomized controlled trials.

#### 4.4.4 Population heterogeneity and precision interventions

Ethnic and geographic differences in gut microbiota characteristics are evident. For example, the HELIUS cohort study (*n* = 4,672) found that gut microbiota explained 4.8% of blood pressure variation in Dutch participants, but only 0.8 and 0.7% in South Asian and African descent participants, respectively (*p* < 0.001). This disparity is linked to genetic factors (such as polymorphisms in the SCFA transporter gene SLC5A8) and dietary habits (such as high carbohydrate intake among South Asians) (Verhaar et al., 2020). National collaboration networks and a nine-year follow-up of 13,000 Chinese adults showed that a traditional southern Chinese diet—rich in rice, vegetables, and fish—significantly reduced cardiovascular risk, likely due to the low-fat, low-glycemic properties of rice, which reduce oxidative stress and insulin resistance (Shi and Ganji, 2020). China’s research emphasizes population cohorts (e.g., cluster #3), while the United States focuses on mechanistic studies (e.g., cluster #0). Across Asia, gut microbiota composition is strongly influenced by geography and diet. For instance, people in southern China who consume high-fiber diets (rice and vegetables) have higher levels of Blautia, which is positively associated with serum threonine; threonine may lower diastolic blood pressure by improving endothelial function (Zhang J. et al., 2024).

Age-stratified research on microbiota interventions reveals further specificity. In childhood hypertension, certain bacterial genera, such as Blautia, have been shown to increase SCFA (e.g., acetate) production, reduce inflammatory markers and visceral fat, and thereby indirectly lower blood pressure. Inulin supplementation can increase Blautia abundance and improve metabolic health in children (Wang J. G. et al., 2023; Chanda et al., 2024). Among older adults, there is significant interaction between gut microbiota and medications. Luo et al. found that hydrochlorothiazide increases Enterobacteriaceae abundance and LPS levels, activating the TLR4 pathway and macrophage polarization, which leads to metabolic disturbances (Luo et al., 2023). Disease subtype–oriented precision interventions have also been investigated. Portal hypertension (cluster #4, silhouette value 0.811) is characterized by increased Proteobacteria; their endotoxin LPS, via TLR4, raises portal pressure. Bajaj et al. found higher Proteobacteria in cirrhosis, correlating with inflammatory markers (IL-6, TNF-α), suggesting that endotoxemia worsens portal hypertension (Bajaj, 2016). Katsuya et al. reported that reduced diversity and increased Proteobacteria, specifically *E. coli* and Klebsiella, are associated with higher portal pressure and act via LPS/TLR4 to promote liver inflammation and fibrosis (Toshida et al., 2023). A 2022 meta-analysis of six RCTs showed that microbiota-targeted therapy, including rifaximin, reduced HVPG by 1.22 mmHg, implying that rifaximin may lower portal pressure by reducing Proteobacteria (Zhang and Gao, 2022). Pulmonary arterial hypertension (an emerging keyword for 2022–2025, strength 4.16) involves hypoxia-induced dysbiosis; studies show that antibiotics can reduce right ventricular pressure in rat models by modulating gut microbiota (Moutsoglou, 2022). Wang et al. identified a key role for gut-derived TMAO in remodeling pulmonary arteries, and inhibiting TMAO production alleviates vascular lesions, offering a novel therapeutic direction (Huang et al., 2022).

Supporting data indicate that China (918 publications) and the United States (676) account for 56.4% of global articles, while India (113) and Brazil (89)—regions with a high hypertension burden—remain underrepresented, creating a gap in heterogeneity data. Keyword cluster analysis (Figure 9) shows that “portal hypertension” (cluster #4) and “blood pressure” (cluster #9) form independent modules, emphasizing the importance of disease subtype research.

This review proposes a three-tiered framework for precision microbiota interventions in hypertension. First, at the ethnic adaptation level, Asian populations appear to benefit most from high-fiber diets, supporting the concept of ethnicity-specific dietary interventions. Second, age-stratified interventions suggest that inulin-induced increases in Blautia may help control childhood hypertension through SCFA production, reduction of inflammation, and viscera fat (Wu et al., 2023; Sun et al., 2025). Third, disease subtype–oriented approaches show that rifaximin can lower hepatic venous pressure gradient (HVPG) in portal hypertension by reducing Proteobacteria, while Akkermansia transplantation can decrease pulmonary arterial pressure in animal models, demonstrating microbiota-based potential for specific hypertension types. Future research faces three main challenges: (1) Data gaps—studies from Africa, South Asia, and other high-burden regions represent only 6% of the global literature, limiting population heterogeneity data; (2) Technical challenges—integrating metagenomics and metabolomics is necessary to unravel personalized microbiome networks (e.g., cluster #7); (3) Clinical translation—large-scale randomized trials are needed to confirm long-term safety and efficacy.

#### 4.4.5 Methodological innovations and emerging trends

Advances in causal inference are exemplified by the application of Mendelian randomization (MR), which uses genetic variants as instrumental variables to mimic randomized controlled trials, thereby minimizing confounding in observational studies. The foundational methodology was established by Smith and Ebrahim (2003). Recent MR studies have demonstrated significant bidirectional causal relationships between the gut microbiota and hypertension and its complications. Certain genera, such as *Clostridium innocuum* and Anaerostipes, are associated with increased hypertension risk, whereas others like Phascolarctobacterium and Allisonella are protective (Li Y. et al., 2023). Notably, each unit increase in Victivallis abundance raises hypertension risk by 8% (OR = 1.08) (Miao et al., 2025). Conversely, hypertension can alter gut microbial composition, lowering protective genera such as Alistipes, thus creating a vicious cycle (Li Y. et al., 2023). Mechanistically, these effects are mediated by microbial metabolites (e.g., SCFAs), inflammatory pathways (e.g., IL-1R2 mediates 14.07% of the effect of *C. innocuum*), and the neuroendocrine axis (Miao et al., 2025). In terms of complications, Lactobacillus and Actinobacteria are linked to higher pulmonary arterial hypertension risk (OR = 2.446 and 3.406), while Ruminococcaceae UCG004 reduces this risk by 59% (OR = 0.407) (Zhang C. et al., 2024). These findings highlight the promise of the gut microbiome as a novel biomarker and therapeutic target for hypertension, supporting probiotic, dietary, or fecal transplant interventions. Burst analysis in this study (Figure 10) revealed that “Mendelian randomization” had the highest burst strength (8.01) during 2023–2025, underscoring its rapid emergence as a leading research method.

The integration of multi-omics and artificial intelligence (AI) is driving the field toward precision medicine. Combined metagenomic and metabolomic analyses enable identification of microbial gene functions and host metabolites, uncovering complex networks connecting the microbiome, metabolites, and blood pressure. For example, Li et al. demonstrated that the gut microbiota influences blood pressure in salt-sensitive hypertension via the tryptophan–indole pathway; metagenomic data revealed increased indole-producing bacteria (e.g., Escherichia-Shigella) in hypertensive rats, along with higher serum indole and vascular inflammation markers (IL-12/IL-10 ratio) (Bardhan et al., 2024). Mathilde Pédrot et al. showed that high-fat diets upregulate intestinal IDO expression, shifting tryptophan metabolism toward the kynurenine pathway, reducing microbial indole metabolites by 40–60% and tripling serum kynurenine. This disrupts the gut barrier and increases inflammation, doubling T-cell infiltration in atherosclerotic plaques. IDO knockout worsened lesions, while indole supplementation restored barrier function and reduced plaque by 35% (Chajadine et al., 2024).

For AI-driven precision prediction, Zeevi et al. (2015) pioneered a machine learning algorithm integrating microbiome, dietary, and clinical data (*n* = 800) to predict postprandial glycemia (AUC = 0.92), a model now extended to blood pressure prediction (Zeevi et al., 2015). The PICRUSt2 tool further predicts microbial metabolic pathways (such as SCFA synthesis) (Douglas et al., 2020). In a study of 51 hypertensive and 51 normotensive children, PICRUSt2 predicted significant enrichment of “cofactor and vitamin metabolism” pathways (including vitamin B synthesis) in hypertensive children’s microbiota. Increased abundance of vitamin B1, B2, and B6 biosynthesis genes (KEGG pathways ko00740, ko00750) was strongly associated with dyslipidemia (high TG, low HDL-C) and reduced sleep, distinguishing hypertensive from normotensive children with an AUC of 0.955 when combined with microbial markers and BMI (Sun et al., 2025).

Network medicine and ecological interaction analyses are providing new paradigms for understanding microbiota–hypertension mechanisms. Co-abundance network analysis, using Spearman correlation, constructs interaction networks to identify functional modules—moving beyond single-taxon studies. Liu et al. found that Ruminococcaceae genera (e.g., UCG-002, UCG-013), even without differential abundance, modulate bile acid metabolism genes through ecological interactions, altering bile acid composition and impacting blood pressure. This mechanism and its clinical relevance were validated in a large multi-regional prospective cohort (*n* = 6,999) (Liu et al., 2025). In terms of host–microbe molecular interactions, cluster #7 (silhouette value 0.816) highlights the use of molecular docking for virtual screening of potential intervention targets (Drakontaeidi and Pontiki, 2023).

Supporting data show that artificial intelligence had a burst strength of 5.32 from 2019 to 2025 (Figure 10), highlighting the growing role of AI. “Network pharmacology” has also seen frequent mention in cluster #7 since 2020, underpinning the rise of network medicine. Keyword clustering (Figure 9) reveals that the “gut microbiota” cluster (#7) includes “molecular docking” and “network pharmacology,” reflecting trends in technological convergence, while the “cardiovascular disease” cluster (#6) is closely related to “precision nutrition,” emphasizing the need for individualized interventions.

Methodological advances in this field can be characterized in three phases. The first is the causal inference phase, exemplified by Mendelian randomization, which addresses confounding from diet and genetics; for example, Heianza et al. (2017) confirmed a causal link between microbial choline metabolism genes and hypertension risk using this method. The second phase is multi-omics integration, combining metagenomic, metabolomic, and AI-based prediction to unravel microbiome–host interactions, pioneered by Zeevi et al.’s (2015) machine learning models. The third phase adopts an ecological perspective, using co-abundance network analysis to reveal the ecological contributions of taxa that do not differ in abundance between groups.

## 5 Strengths and limitations

This study has several significant limitations. First, due to the data format limitations of VOSviewer and CiteSpace, our literature search was restricted to the Web of Science Core Collection (WOSCC). Although WOSCC covers clinical medicine extensively, it may overlook interdisciplinary studies, particularly those bridging microbiology and cardiovascular science. Consequently, relevant studies in other databases or in non-English languages may have been missed. Future studies should incorporate databases like PubMed, Scopus, and Embase to minimize selection bias and improve coverage. This integration would enhance the methodological robustness of future research. Second, since WOSCC mainly indexes high-impact English-language journals, non-English studies, such as smaller clinical research from Asia or South America, may be excluded. This exclusion leads to limited geographic representation. For instance, Table 1 indicates that research from high-prevalence regions such as India and Brazil comprises only 12.3% of the total. We recommend developing bibliometric tools with multilingual analysis capabilities, such as CiteSpace plugins integrated with translation APIs, to increase inclusivity of studies from various regions. Third, the presence of synonyms among keywords (e.g., “hypertension” vs. “high blood pressure,” or “gut microbiota” vs. “intestinal microbiome”) may have caused minor overlaps during keyword clustering. However, all clustering modules in our analysis had Silhouette scores greater than 0.5. Key modules, such as #0 (short-chain fatty acids) and #5 (trimethylamine N-oxide), achieved scores above 0.64, indicating high reliability and consistency. These minor overlaps may require some adjustments, but they do not compromise the clarity of the main topic clusters. Future research can further improve clustering accuracy through manual validation.

## 6 Conclusion

Utilizing bibliometric analysis with CiteSpace and VOSviewer, this study systematically examined the research landscape and evolving trends in the field of hypertension and the gut microbiota. The results indicate that China (918 publications) and the United States (676 publications) serve as dual centers, leading both mechanistic and translational research on the microbiome–metabolite–blood pressure axis and forming an international collaboration network primarily led by these two countries with contributions from multiple others. Research hotspots have evolved through three distinct stages: initial focus on the gut-liver axis (2000–2013), followed by exploration of metabolic disorders and hypertension (2014–2019), and more recently, the rise of precision microbiome modulation (2020–2025). Key research focuses now include metabolites such as short-chain fatty acids and trimethylamine N-oxide, as well as functional genera like *Akkermansia muciniphila*. Techniques such as Mendelian randomization and multi-omics integration have advanced the field toward understanding causal mechanisms and developing personalized interventions. Three main research modules—metabolic regulation, associations with complications, and intervention strategies—have become clearly defined. Studies on the microbiome’s role in specific disease subtypes, such as portal hypertension and pulmonary arterial hypertension, are now emerging as new frontiers. Intervention strategies, including rifaximin-mediated suppression of Proteobacteria and transplantation of Akkermansia to ameliorate hypoxic injury, show promising clinical potential. Methodologically, advances such as integrated metagenomic and metabolomic analyses, AI-driven predictive modeling, and co-abundance network analysis are driving comprehensive understanding of the interactions among the microbiome, host, and environment. The quantitative evolution model developed in this study highlights the key developmental pathways in the field and provides a theoretical foundation for overcoming current barriers in clinical translation. In the future, there is a need to strengthen cohort studies in regions such as Africa and South Asia, integrate multi-omics and network medicine approaches, and generate more clinical evidence for microbiome-targeted therapies, ultimately paving new pathways for the precise prevention and control of hypertension.

## Data Availability

The original contributions presented in the study are included in the article/supplementary material, further inquiries can be directed to the corresponding author.

## References

[ref1] Al Khodor S. Reichert B. Shatat I. F. (2017). The microbiome and blood pressure: can microbes regulate our blood pressure? Front. Pediatr. 5:138. doi: 10.3389/fped.2017.00138, PMID: PMC5474689 28674682

[ref2] Azuma N. Saito Y. Nishijima T. Aoki R. Nishihira J. (2023). Effect of daily ingestion of bifidobacterium and dietary fiber on vascular endothelial function: a randomized, double-blind, placebo-controlled, parallel-group comparison study. *Biosci., Biotechnol*. Biochemist 88, 86–96. doi: 10.1093/bbb/zbad148, PMID: 37849220

[ref3] Bajaj J. S. (2016). Review article: potential mechanisms of action of rifaximin in the management of hepatic encephalopathy and other complications of cirrhosis. Aliment. Pharmacol. Ther. 43, 11–26. doi: 10.1111/apt.13435, PMID: 26618922

[ref4] Bao Y. Deng Z. Wang Y. Kim H. Armengol V. D. Acevedo F. . (2019). Using machine learning and natural language processing to review and classify the medical literature on cancer susceptibility genes. JCO Clin. Cancer Inform. 3, 1–9. doi: 10.1200/CCI.19.00042, PMID: 31545655 PMC6873946

[ref5] Bardhan P. Mei X. Lai N. K. Mell B. Tummala R. Aryal S. . (2024). Salt-responsive gut microbiota induces sex-specific blood pressure changes. Circ. Res. 135, 1122–1137. doi: 10.1161/CIRCRESAHA.124.325056, PMID: 39440438 PMC11905770

[ref6] Bridgeman S. C. Northrop W. Melton P. E. Ellison G. C. Newsholme P. Mamotte C. D. S. (2020). Butyrate generated by gut microbiota and its therapeutic role in metabolic syndrome. Pharmacol. Res. 160:105174. doi: 10.1016/j.phrs.2020.105174, PMID: 32860943

[ref7] Carey R. M. Calhoun D. A. Bakris G. L. Brook R. D. Daugherty S. L. Dennison-Himmelfarb C. R. . (2018). Resistant hypertension: detection, evaluation, and management: a scientific statement from the American heart association. Hypertens. (Dallas Tex,: 1979) 72, e53–e90. doi: 10.1161/HYP.0000000000000084, PMID: 30354828 PMC6530990

[ref8] Chajadine M. Laurans L. Radecke T. Mouttoulingam N. Al-Rifai R. Bacquer E. . (2024). Harnessing intestinal tryptophan catabolism to relieve atherosclerosis in mice. Nat. Commun. 15:6390. doi: 10.1038/s41467-024-50807-x, PMID: 39080345 PMC11289133

[ref9] Chanda W. Jiang H. Liu S.-J. (2024). The ambiguous correlation of blautia with obesity: a systematic review. Microorganisms 12:1768. doi: 10.3390/microorganisms12091768, PMID: 39338443 PMC11433710

[ref10] Chen C. (2006). CiteSpace II: detecting and visualizing emerging trends and transient patterns in scientific literature. J. Am. Soc. Inf. Sci. 57, 359–377. doi: 10.1002/asi.20317

[ref11] Chen C. (2017). Science mapping: a systematic review of the literature. J. Data Inf. Sci. 2, 1–40. doi: 10.1515/jdis-2017-0006

[ref12] Chen C.-M. Wu C.-C. Huang C.-L. Chang M.-Y. Cheng S.-H. Lin C.-T. . (2022). *Lactobacillus plantarum* PS128 promotes intestinal motility, mucin production, and serotonin signaling in mice. Probiotics Antimicrob. Proteins 14, 535–545. doi: 10.1007/s12602-021-09814-3, PMID: 34327633 PMC9076750

[ref13] Depommier C. Everard A. Druart C. Plovier H. Van Hul M. Vieira-Silva S. . (2019). Supplementation with *akkermansia muciniphila* in overweight and obese human volunteers: a proof-of-concept exploratory study. Nat. Med. 25, 1096–1103. doi: 10.1038/s41591-019-0495-2, PMID: PMC6699990 31263284

[ref14] Differding M. Hivert M.-F. Doyon M. Bouchard L. Perron P. Guérin R. . (2020). Gut microbiome composition is associated with blood pressure in mother-child pairs 5 years after birth. Curr. Dev. Nutr. 4:nzaa062_012. doi: 10.1093/cdn/nzaa062_012

[ref15] Dong Y. Wang P. Jiao J. Yang X. Chen M. Li J. (2022). Antihypertensive therapy by ACEI/ARB is associated with intestinal flora alterations and metabolomic profiles in hypertensive patients. Front. Cell Dev. Biol. 10:861829. doi: 10.3389/fcell.2022.861829, PMID: PMC8986158 35399511

[ref16] Douglas G. M. Maffei V. J. Zaneveld J. R. Yurgel S. N. Brown J. R. Taylor C. M. . (2020). PICRUSt2 for prediction of metagenome functions. Nat. Biotechnol. 38, 685–688. doi: 10.1038/s41587-020-0548-6, PMID: PMC7365738 32483366

[ref17] Drakontaeidi A. Pontiki E. (2023). A review on molecular docking on HDAC isoforms: novel tool for designing selective inhibitors. Pharmaceuticals 16:1639. doi: 10.3390/ph16121639, PMID: PMC10746130 38139766

[ref18] Duarte Luiz J. Manassi C. Magnani M. da Cruz A. G. Pimentel T. C. Verruck S. (2025). *Lactiplantibacillus plantarum* as a promising adjuvant for neurological disorders therapy through the brain-gut axis and related action pathways. Crit. Rev. Food Sci. Nutr. 65, 715–727. doi: 10.1080/10408398.2023.2280247, PMID: 37950651

[ref19] Durgan D. J. Ganesh B. P. Cope J. L. Ajami N. J. Phillips S. C. Petrosino J. F. . (2016). Role of the gut microbiome in obstructive sleep apnea-induced hypertension. Hypertension 67, 469–474. doi: 10.1161/HYPERTENSIONAHA.115.06672, PMID: PMC4713369 26711739

[ref20] Efremova I. Maslennikov R. Poluektova E. Medvedev O. Kudryavtseva A. Krasnov G. . (2024). Gut microbiota and biomarkers of endothelial dysfunction in cirrhosis. Int. J. Mol. Sci. 25:14. doi: 10.3390/ijms25041988, PMID: PMC10888218 38396668

[ref21] Georgoulis M. Damigou E. Derdelakou E. Kosti R. I. Chrysohoou C. Barkas F. . (2024). Adherence to the mediterranean diet and 20-year incidence of hypertension: the ATTICA prospective epidemiological study (2002-2022). Eur. J. Clin. Nutr. 78, 630–638. doi: 10.1038/s41430-024-01440-w, PMID: 38605190

[ref22] He G. Cao Y. Wang H. Lv X. (2023). Causal effects of gut microbiome on hypertension: a mendelian randomization study. Front. Microbiol. 14:1276050. doi: 10.3389/fmicb.2023.1276050, PMID: 38088967 PMC10712025

[ref9001] Heianza Y. Ma W. Manson J. E. Rexrode K. M. Qi L. (2017). Gut microbiota metabolites and risk of major adverse cardiovascular disease events and death: a systematic review and meta-analysis of prospective studies. J Am Heart Assoc. 6, e004947. doi: 10.1161/JAHA.116.004947 28663251 PMC5586261

[ref23] Huang Y. Lin F. Tang R. Bao C. Zhou Q. Ye K. . (2022). Gut microbial metabolite trimethylamine N-oxide aggravates pulmonary hypertension. Am. J. Respir. Cell Mol. Biol. 66, 452–460. doi: 10.1165/rcmb.2021-0414OC, PMID: 35100519

[ref24] Huc T. Drapala A. Gawrys M. Konop M. Bielinska K. Zaorska E. . (2018). Chronic, low-dose TMAO treatment reduces diastolic dysfunction and heart fibrosis in hypertensive rats. Am. J. Physiol. Heart Circ. Physiol. 315, H1805–H1820. doi: 10.1152/ajpheart.00536.2018, PMID: 30265149

[ref25] Jama H. A. Snelson M. Schutte A. E. Muir J. Marques F. Z. (2024). Recommendations for the use of dietary fiber to improve blood pressure control. Hypertens (dallas Tex,: 1979) 81, 1450–1459. doi: 10.1161/HYPERTENSIONAHA.123.22575 38586958

[ref26] Jaworska K. Huc T. Samborowska E. Dobrowolski L. Bielinska K. Gawlak M. . (2017). Hypertension in rats is associated with an increased permeability of the colon to TMA, a gut bacteria metabolite. PLoS One 12:e0189310. doi: 10.1371/journal.pone.0189310, PMID: 29236735 PMC5728578

[ref27] Kasahara K. Krautkramer K. A. Org E. Romano K. A. Kerby R. L. Vivas E. I. . (2018). Interactions between roseburia intestinalis and diet modulate atherogenesis in a murine model. Nat. Microbiol. 3, 1461–1471. doi: 10.1038/s41564-018-0272-x, PMID: PMC6280189 30397344

[ref28] Kaye D. M. Shihata W. A. Jama H. A. Tsyganov K. Ziemann M. Kiriazis H. . (2020). Deficiency of prebiotic fiber and insufficient signaling through gut metabolite-sensing receptors leads to cardiovascular disease. Circulation 141, 1393–1403. doi: 10.1161/CIRCULATIONAHA.119.043081, PMID: 32093510

[ref29] Khalesi S. Sun J. Buys N. Jayasinghe R. (2014). Effect of probiotics on blood pressure: a systematic review and meta-analysis of randomized, controlled trials. Hypertension 64, 897–903. doi: 10.1161/HYPERTENSIONAHA.114.03469, PMID: 25047574

[ref30] Kimura I. Inoue D. Maeda T. Hara T. Ichimura A. Miyauchi S. . (2011). Short-chain fatty acids and ketones directly regulate sympathetic nervous system via G protein-coupled receptor 41 (GPR41). Proc. Natl. Acad. Sci. USA 108, 8030–8035. doi: 10.1073/pnas.1016088108, PMID: 21518883 PMC3093469

[ref31] Kimura I. Ozawa K. Inoue D. Imamura T. Kimura K. Maeda T. . (2013). The gut microbiota suppresses insulin-mediated fat accumulation via the short-chain fatty acid receptor GPR43. Nat. Commun. 4:1829. doi: 10.1038/ncomms2852, PMID: PMC3674247 23652017

[ref32] Koeth R. A. Wang Z. Levison B. S. Buffa J. A. Org E. Sheehy B. T. . (2013). Intestinal microbiota metabolism of L-carnitine, a nutrient in red meat, promotes atherosclerosis. Nat. Med. 19, 576–585. doi: 10.1038/nm.3145, PMID: 23563705 PMC3650111

[ref33] Kong L. Zhao Q. Jiang X. Hu J. Jiang Q. Sheng L. . (2024). Trimethylamine N-oxide impairs β-cell function and glucose tolerance. Nat. Commun. 15:2526. doi: 10.1038/s41467-024-46829-0, PMID: 38514666 PMC10957989

[ref34] Lan Y. Ning K. Ma Y. Zhao J. Ci C. Yang X. . (2022). High-density lipoprotein cholesterol as a potential medium between depletion of lachnospiraceae genera and hypertension under a high-calorie diet. Microbiol. Spectr. 10:e0234922. doi: 10.1128/spectrum.02349-22, PMID: 36250859 PMC9769594

[ref35] Li Y. Fu R. Li R. Zeng J. Liu T. Li X. . (2023). Causality of gut microbiome and hypertension: a bidirectional mendelian randomization study. Front. Cardiovasc. Med. 10:1167346. doi: 10.3389/fcvm.2023.1167346, PMID: 37215554 PMC10192878

[ref36] Li X. S. Obeid S. Klingenberg R. Gencer B. Mach F. Räber L. . (2017). Gut microbiota-dependent trimethylamine N-oxide in acute coronary syndromes: a prognostic marker for incident cardiovascular events beyond traditional risk factors. Eur. Heart J. 38, 814–824. doi: 10.1093/eurheartj/ehw582, PMID: 28077467 PMC5837488

[ref37] Li J. Zhong Y. Bai J. Chen S. Cai J. Wu S. . (2023). Composition and functional capacity of gut microbes are associated with arterial stiffness: a prospective study. Cardiol. Discov. 3, 102–111. doi: 10.1097/CD9.0000000000000085

[ref38] Li Y. Zhou E. Yu Y. Wang B. Zhang L. Lei R. . (2024). Butyrate attenuates cold-induced hypertension via gut microbiota and activation of brown adipose tissue. Sci. Total Environ. 943:173835. doi: 10.1016/j.scitotenv.2024.173835, PMID: 38851345

[ref39] Liu X.-Y. Li J. Zhang Y. Fan L. Xia Y. Wu Y. . (2022). Kidney microbiota dysbiosis contributes to the development of hypertension. Gut Microbes 14:2143220. doi: 10.1080/19490976.2022.2143220, PMID: 36369946 PMC9662196

[ref40] Liu L. Zhou Q. Xu T. Deng Q. Sun Y. Fu J. . (2025). Non-differential gut microbes contribute to hypertension and its severity through co-abundances: a multi-regional prospective cohort study. iMeta 4:e268. doi: 10.1002/imt2.268, PMID: PMC11865328 40027484

[ref41] Luo J.-Q. Ren H. Chen M.-Y. Zhao Q. Yang N. Liu Q. . (2023). Hydrochlorothiazide-induced glucose metabolism disorder is mediated by the gut microbiota via LPS-TLR4-related macrophage polarization. Iscience 26:107130. doi: 10.1016/j.isci.2023.107130, PMID: 37456847 PMC10338205

[ref42] Mahgoup E. M. (2025). “Gut microbiota as a therapeutic target for hypertension: challenges and insights for future clinical applications” “gut microbiota and hypertension therapy”. Curr. Hypertens. Rep. 27:14. doi: 10.1007/s11906-025-01331-w, PMID: 40261509

[ref43] Miao C. Xu X. Huang S. Kong L. He Z. Wang Y. . (2025). The causality between gut microbiota and hypertension and hypertension-related complications: a bidirectional two-sample mendelian randomization analysis. Hell. J. Cardiol. 83, 38–50. doi: 10.1016/j.hjc.2024.02.002, PMID: 38336261

[ref44] Moutsoglou D. M. (2022). 2021 American thoracic society BEAR cage winning proposal: microbiome transplant in pulmonary arterial hypertension. Am. J. Respir. Crit. Care Med. 205, 13–16. doi: 10.1164/rccm.202108-1833ED, PMID: PMC8865595 34758276

[ref45] NCD Risk Factor Collaboration (2021). Worldwide trends in hypertension prevalence and progress in treatment and control from 1990 to 2019: a pooled analysis of 1201 population-representative studies with 104 million participants. Lancet (Lond. Engl.) 398, 957–980. doi: 10.1016/S0140-6736(21)01330-1, PMID: 34450083 PMC8446938

[ref46] Pelletier C. C. Croyal M. Ene L. Aguesse A. Billon-Crossouard S. Krempf M. . (2019). Elevation of trimethylamine-N-oxide in chronic kidney disease: contribution of decreased glomerular filtration rate. Toxins 11:635. doi: 10.3390/toxins11110635, PMID: 31683880 PMC6891811

[ref47] Pluznick J. L. Protzko R. J. Gevorgyan H. Peterlin Z. Sipos A. Han J. . (2013). Olfactory receptor responding to gut microbiota-derived signals plays a role in renin secretion and blood pressure regulation. Proc. Natl. Acad. Sci. USA 110, 4410–4415. doi: 10.1073/pnas.1215927110, PMID: 23401498 PMC3600440

[ref48] Prisco S. Z. Oliveira S. D. Weir E. K. Thenappan T. Al Ghouleh I. (2025). Gut microbiome in pulmonary arterial hypertension—an emerging frontier. Infect. Dis. Rep. 17:66. doi: 10.3390/idr17030066, PMID: 40559197 PMC12193523

[ref49] Qi J. You T. Li J. Pan T. Xiang L. Han Y. . (2018). Circulating trimethylamine N-oxide and the risk of cardiovascular diseases: a systematic review and meta-analysis of 11 prospective cohort studies. J. Cell. Mol. Med. 22, 185–194. doi: 10.1111/jcmm.13307, PMID: 28782886 PMC5742728

[ref50] Ren Y. Chen L. Guo R. Ma S. Li S. Zhang Y. . (2024). Altered gut mycobiome in patients with end-stage renal disease and its correlations with serum and fecal metabolomes. J. Transl. Med. 22:202. doi: 10.1186/s12967-024-05004-1, PMID: 38403655 PMC10894479

[ref51] Renying X. U. Yiquan Z. Xiaomin Z. Zhiqi C. Yanping W. (2018). Relationship between percent body fat and hypertension in children and adolescents. J. Clin. Pediatr. Available at: http://en.cnki.com.cn/Article_en/CJFDTOTAL-LCAK201807002.htm (Accessed July 1, 2025).

[ref52] Shi Z. Ganji V. (2020). Dietary patterns and cardiovascular disease risk among Chinese adults: a prospective cohort study. Eur. J. Clin. Nutr. 74, 1725–1735. doi: 10.1038/s41430-020-0668-6, PMID: 32506113

[ref53] Shi H. Li X. Hou C. Chen L. Zhang Y. Li J. (2023). Effects of pomegranate peel polyphenols combined with inulin on gut microbiota and serum metabolites of high-fat-induced obesity rats. J. Agric. Food Chem. 71, 5733–5744. doi: 10.1021/acs.jafc.3c01014, PMID: 36996454

[ref54] Singh A. B. BinKraemer F. B. Xu Y. Zhang Y. Liu J. (2018). Farnesoid X receptor activation by obeticholic acid elevates liver low-density lipoprotein receptor expression by mRNA stabilization and reduces plasma low-density lipoprotein cholesterol in mice. Arterioscler. Thromb. Vasc. Biol. 38, 2448–2459. doi: 10.1161/ATVBAHA.118.311122 30354208 PMC6206879

[ref55] Smith G. D. Ebrahim S. (2003). “Mendelian randomization”: can genetic epidemiology contribute to understanding environmental determinants of disease? Int. J. Epidemiol. 32, 1–22. doi: 10.1093/ije/dyg070, PMID: 12689998

[ref56] Sun D. Xiang H. Yan J. He L. (2022). Intestinal microbiota: a promising therapeutic target for hypertension. Front. Cardiovasc. Med. 9:970036. doi: 10.3389/fcvm.2022.970036, PMID: 36457803 PMC9705378

[ref57] Sun J. Yang L. Ma C. Yang L. Zhao M. Magnussen C. G. . (2025). Alteration of gut microbiota associated with hypertension in children. BMC Microbiol. 25:282. doi: 10.1186/s12866-025-03999-1, PMID: 40340772 PMC12060425

[ref58] Tang W. H. W. Wang Z. Kennedy D. J. Wu Y. Buffa J. A. Agatisa-Boyle B. . (2015). Gut microbiota-dependent trimethylamine N-oxide (TMAO) pathway contributes to both development of renal insufficiency and mortality risk in chronic kidney disease. Circ. Res. 116, 448–455. doi: 10.1161/CIRCRESAHA.116.305360, PMID: 25599331 PMC4312512

[ref59] Tilves C. Yeh H.-C. Maruthur N. Juraschek S. P. Miller E. White K. . (2022). Increases in circulating and fecal butyrate are associated with reduced blood pressure and hypertension: results from the SPIRIT trial. J. Am. Heart Assoc. 11:e024763. doi: 10.1161/JAHA.121.024763, PMID: 35730613 PMC9333372

[ref60] Toshida K. Itoh S. Kosai-Fujimoto Y. Ishikawa T. Nakayama Y. Tsutsui Y. . (2023). Association of gut microbiota with portal vein pressure in patients with liver cirrhosis undergoing living donor liver transplantation. JGH Open 7, 982–989. doi: 10.1002/jgh3.13018, PMID: 38162858 PMC10757484

[ref61] van Eck N. J. Waltman L. (2010). Software survey: VOSviewer, a computer program for bibliometric mapping. Scientometrics 84, 523–538. doi: 10.1007/s11192-009-0146-3, PMID: 20585380 PMC2883932

[ref62] Verhaar B. J. H. Collard D. Prodan A. Levels J. H. M. Zwinderman A. H. Bäckhed F. . (2020). Associations between gut microbiota, faecal short-chain fatty acids, and blood pressure across ethnic groups: the HELIUS study. Eur. Heart J. 41, 4259–4267. doi: 10.1093/eurheartj/ehaa704, PMID: 32869053 PMC7724641

[ref63] Wang Q. Dai H. Hou T. Hou Y. Wang T. Lin H. . (2023). Dissecting causal relationships between gut microbiota, blood metabolites, and stroke: a mendelian randomization study. J. Stroke 25, 350–360. doi: 10.5853/jos.2023.00381, PMID: 37813672 PMC10574297

[ref64] Wang H. Luo Q. Ding X. Chen L. Zhang Z. (2022). Trimethylamine N-oxide and its precursors in relation to blood pressure: a mendelian randomization study. Front. Cardiovasc. Med. 9:922441. doi: 10.3389/fcvm.2022.922441, PMID: 35935641 PMC9354484

[ref65] Wang P. Shen Y. Yan K. Wang S. Jiao J. Chi H. . (2025). CKD patients comorbid with hypertension are associated with imbalanced gut microbiome. Iscience 28:111766. doi: 10.1016/j.isci.2025.111766, PMID: 39911351 PMC11795142

[ref66] Wang M. Tang W. H. W. Li X. S. de Oliveira Otto M. C. Lee Y. Lemaitre R. N. . (2024). The gut microbial metabolite trimethylamine N -oxide, incident CKD, and kidney function decline. J. Am. Soc. Nephrol. 35, 749–760. doi: 10.1681/ASN.0000000000000344, PMID: 38593157 PMC11164118

[ref67] Wang J.-G. Zhang W. Li Y. Liu L. (2023). Hypertension in China: epidemiology and treatment initiatives. Nat. Rev. Cardiol. 20, 531–545. doi: 10.1038/s41569-022-00829-z, PMID: 36631532

[ref68] Wu X.-R. Chen Z.-Z. Dong X.-L. Zhao Q.-P. Cai J. (2023). A novel symbiotic formulation reduces obesity and concomitant metabolic syndrome in rats by raising the relative abundance of blautia. Nutrients 15:956. doi: 10.3390/nu15040956, PMID: 36839314 PMC9960556

[ref69] Yang T. Santisteban M. M. Rodriguez V. Li E. Ahmari N. Carvajal J. M. . (2015). Gut dysbiosis is linked to hypertension. Hypertension 65, 1331–1340. doi: 10.1161/HYPERTENSIONAHA.115.05315, PMID: 25870193 PMC4433416

[ref70] Yu H. Chai X. Geng W.-C. Zhang L. Ding F. Guo D.-S. . (2021). Facile and label-free fluorescence strategy for evaluating the influence of bioactive ingredients on FMO3 activity via supramolecular host-guest reporter pair. Biosens. Bioelectron. 192:113488. doi: 10.1016/j.bios.2021.113488, PMID: 34265522

[ref71] Zaharuddin A. M. Muslim A. Aazmi S. Idorus M. Y. Almabhouh F. A. Lim S. Y. . (2025). Probiotic *lactobacillus rhamnosus* GG alleviates prehypertension and restores gut health and microbiota in NaCl-induced prehypertensive rats. Probiotics Antimicrob. Proteins. doi: 10.1007/s12602-025-10536-z 40254701

[ref72] Zeevi D. Korem T. Zmora N. Israeli D. Rothschild D. Weinberger A. . (2015). Personalized nutrition by prediction of glycemic responses. Cell 163, 1079–1094. doi: 10.1016/j.cell.2015.11.001, PMID: 26590418

[ref73] Zhang H. Gao J. (2022). Antibiotics and probiotics on hepatic venous pressure gradient in cirrhosis: a systematic review and a meta-analysis. PLoS One 17:e0273231. doi: 10.1371/journal.pone.0273231, PMID: 36040984 PMC9426916

[ref74] Zhang J. Qi H. Li M. Wang Z. Jia X. Sun T. . (2024). Diet mediate the impact of host habitat on gut microbiome and influence clinical indexes by modulating gut microbes and serum metabolites. Adv Sci (Weinh.) 11:e2310068. doi: 10.1002/advs.202310068 38477427 PMC11109649

[ref75] Zhang C. Xi Y. Zhang Y. He P. Su X. Li Y. . (2024). Causal effects between gut microbiota and pulmonary arterial hypertension: a bidirectional mendelian randomization study. Heart Lung 64, 189–197. doi: 10.1016/j.hrtlng.2024.01.002, PMID: 38290183

[ref76] Zhang X. Yin Y. Chen Y. Lin L. Shen S. Fang F. . (2025). Gut microbiota contributes to obstructive sleep apnea-induced hypertension by gut-heart axis in mice. Int. Immunopharmacol. 155:114667. doi: 10.1016/j.intimp.2025.114667, PMID: 40245774

